# Causal relationship between reproductive factors and female bone density: a univariate and multivariate mendelian randomization study

**DOI:** 10.3389/fgene.2024.1393106

**Published:** 2024-09-13

**Authors:** Xiaojing Lin, Yaqi Zuo, Hongbo Hu, Jie Zhou

**Affiliations:** ^1^ Guangdong Medical University, Zhanjiang, China; ^2^ Yuebei People’s Hospital, Guangdong Medical University, Shaoguan, China; ^3^ Yuebei People’s Hospital, Shaoguan, China

**Keywords:** reproductive factors, mendelian randomization, BMD, osteoporosis, female

## Abstract

**Objective:**

Observational studies have found associations between reproductive factors and bone density in women. However, the causal relationships are not well understood. This study aims to investigate whether various reproductive factors are causally related to bone density at different skeletal sites using both univariable and multivariable Mendelian randomization (MR) methods.

**Methods:**

The study incorporated four reproductive factors, namely, age at menarche (AAM), age at first live birth (AFB), age at menopause (ANM), and age at last live birth (ALB), as well as five distinct skeletal sites, including bone mineral density (BMD), heel calcaneus BMD, ultradistal forearm bone mineral density (FA-BMD), lumbar spine bone mineral density (LS-BMD), and femoral neck bone mineral density (FN-BMD). Univariable two-sample MR and multivariable MR analyses were conducted using data from published genome-wide association studies (GWASs). A total of 150 single nucleotide polymorphisms (SNPs) associated with the four reproductive factors were extracted from GWAS databases. The primary statistical analysis method utilized in this study was the inverse variance weighted (IVW) method.

**Results:**

In the univariate MR analysis, we observed causal connections between four reproductive factors and bone density. Specifically, AAM had a significant impact on BMD and heel calcaneus BMD. Age at first live birth was negatively associated with FA-BMD. Age at last live birth showed a negative correlation with BMD and heel calcaneus BMD. ANM exhibited positive associations with BMD, heel calcaneus BMD, FA-BMD, and LS-BMD. Subsequently, we performed a multivariable MR analysis to examine the combined effects of multiple variables, which confirmed the persistence of associations between age at menopause and bone density at various sites. Additionally, we found a negative correlation between age at last live birth and heel calcaneus BMD.

**Conclusion:**

This study offers a fresh perspective on the prevention of osteoporosis in women, explicitly stating that reproductive factors such as early menopause and late childbirth play a significant predictive role in individual bone density decline. Therefore, when developing osteoporosis screening and management protocols, reproductive factors should be included for a more comprehensive guidance of clinical practice.

## 1 Introduction

Osteoporosis is a common systemic skeletal disease and a prevalent condition among elderly women. It is characterized by reduced bone density and altered bone tissue structure, significantly increasing the risk of osteoporotic fractures, commonly occurring at the hip, radius/ulna,vertebra and humerus (Konstantelos et al., 2022). With the aging population, it is predicted that the global elderly population will reach 2.1 billion in the next 25 years (https://www.who.int/news-room/fact-sheets/detail/ageing-and-health), and an increasing number of elderly women will face the burden of osteoporosis. The prevalence of osteoporosis in women aged 50 or above is documented to be 29.9% (Wright et al., 2017). This situation contributes to individual and national healthcare costs, seriously impairs women’s health and quality of life, and may even lead to mortality following fractures (Konstantelos et al., 2022). Reproductive factors, as one of the physiological characteristics in women, have been associated with bone density, but existing studies on this relationship remain controversial (Miglioli et al., 2007; Cho et al., 2012), and the causal relationship is yet to be established.

MR is a research method that utilizes genetic variants as instruments for natural random allocation to evaluate causal relationships between potential factors. Through single nucleotide polymorphisms (SNPs), MR assesses the causal effects of exposure factors on the outcome variables. Univariate MR reduces the impact of confounding factors and determines the causal relationship between the exposure factor and the outcome variable. In combination with multivariable MR, it allows for understanding the interactions and comprehensive effects of multiple included exposure factors, thereby controlling for confounding biases and obtaining more reliable research results.

In this research, we aim to explore the correlation and causal relationship between reproductive factors (AAM, ANM, age at first and last childbirth) and site-specific bone density in women using univariate and multivariable MR methods. By filling the gaps in existing research, we provide a more scientific basis and guidance for early clinical prevention and treatment of osteoporosis and other bone-related diseases.

## 2 Methods

We employed the MR method to investigate the relationship between four reproductive factors and bone density in five different sites in this study. We conducted univariable two-sample MR and multivariable MR analyses. For this analysis, we utilized summary-level data from published genome-wide association studies (GWASs). Approval from appropriate review committees and informed consent from all participants were obtained for all referenced GWAS studies.

### 2.1 Instrumental variable selection

We selected AAM, AFB, ANM, and ALB as the exposure factors for this study. We conducted a search utilizing the IEU database (https://gwas.mrcieu.ac.uk/) and applied a filtration process to identify relevant Single Nucleotide Polymorphisms (SNPs) based on their significance at the genome-wide level (*p* ≤ 5 × 10^−8^). Simultaneously, we excluded SNPs with a linkage disequilibrium value (r2≥0.01) to ensure data accuracy. Using the PhenoScanner database, we eliminated genetic instrumental variables (IVs) related to confounding factors. We excluded weak instrumental variables (IVs) with an F-statistic less than 10. We utilized four distinct reproductive factors and bone mineral density (BMD) at five different sites as the analytical targets, resulting in 20 unique analyses (each reproductive factor corresponding to five BMD analyses). The distinct sets of SNPs for each analysis are enumerated in the Supplementary Table S1 ranging from Supplementary Tables S3–22. Supplementary Table S1 provides a comprehensive summary of the exposure factor GWAS studies, including detailed information.

### 2.2 Data sources related to BMD at different sites

We selected BMD,heel calcaneus BMD,FA-BMD,LS-BMD,FN-BMD as the outcome variables for our study. We performed searches in the IEU database (https://gwas.mrcieu.ac.uk/) for each of these outcome factors and summarized the detailed information from the corresponding GWAS studies in Supplementary Table S2.

### 2.3 Statistical analysis

This study employed a univariable and multivariable MR study design to investigate the impact of reproductive factors on bone density at different sites. Four reproductive factors were selected as exposure variables, and bone density at five different sites were considered as outcome variables. To investigate the effects of reproductive factors on bone density at various sites, we initially performed univariable MR analyses to assess the individual impacts of each reproductive factor. Subsequently, a multivariable MR study was conducted to evaluate the collective influence of these reproductive factors on bone density across different sites.

The analyses were performed utilizing the Two-Sample MR package within the R software environment (version 4.3.2). After harmonizing exposure and outcome data, we performed genotype encoding and allele harmonization for SNPs, excluding those with inconsistent alleles, and excluded all possible palindromic SNPs in sensitivity analysis. Proxy SNPs were not used to replace partially missing instrumental variables since the impact of missing data on the results was minimal.

The inverse variance weighted (IVW) method was primarily used for statistical analysis in this study, and additional analyses including Weighted median, MR-Egger, Weighted mode, and Simple mode were conducted as supplementary and sensitivity analyses (Davies et al., 2019). MR-Egger was utilized to assess the directional pleiotropy of IVs, while the weighted median method provided increased accuracy compared to MR-Egger. For multivariable MR, regression-based IVW was employed for statistical analysis. If there is no clear evidence of directional pleiotropy (*p* > 0.05 for MR-Egger intercept), the IVW method is considered the most accurate approach for estimating causal relationships.

## 3 Results

### 3.1 Univariable MR

We used four reproductive factors (AAM, AFB, ANM, ALB) as exposure variables and studied their effects on BMD, heel calcaneus BMD, FA-BMD, LS-BMD, and FN-BMD as outcome variables through univariable MR analysis. The summarized information is presented in Table 1. The F-statistic values for the instrumental variables exceeded 10, indicating good instrument strength of the SNPs used.

**TABLE 1:** Univariate MR analysis of four different reproductive factors and five different sites of BMD respectively.

Outcome	snps	beta (95%Cl)			P	F
USING AGE AT MENARCHE SNPs						
Bone mineral density	6	−0.06 (−0.10,-0.02)			0.007	64.9
Heel bone mineral density	5	−0.07 (−0.11,-0.02)			0.002	62.9
Ultradistal forearm bone mineral density	6	0.06 (−0.03,0.15)			0.200	65.4
Lumbar spine bone mineral density	4	−0.06 (−0.15,0.03)			0.192	63.1
Femoral neck bone mineral density	4	−0.06 (−0.16,0.04)			0.257	63.0
USING AGE AT FIRST LIVE BIRTH SNPs						
Bone mineral density	32	−0.03 (−0.12,0.06)			0.483	37.7
Heel bone mineral density	32	−0.02 (0.11,0.07)			0.641	37.8
Ultradistal forearm bone mineral density	33	−0.20 (−0.37,-0.02)			0.028	37.6
Lumbar spine bone mineral density	32	0.09 (−0.06,0.23)			0.254	38.0
Femoral neck bone mineral density	32	0.04 (−0.09,0.17)			0.541	38.0
USING AGE AT MENOPAUSE SNPs						
Bone mineral density	93	0.05 (0.02,0.09)			0.003	87.1
Heel bone mineral density	106	0.06 (0.02,0.09)			0.001	88.6
Ultradistal forearm bone mineral density	100	0.10 (0.03,0.16)			0.003	89.3
Lumbar spine bone mineral density	92	0.10 (0.03,0.17)			0.005	75.3
Femoral neck bone mineral density	92	0.05 (0.00,0.11)			0.054	75.3
USING AGE AT LAST LIVE BIRTH SNPs						
Bone mineral density	6	−0.25 (−0.49,-0.01)			0.041	40.3
Heel bone mineral density	5	−0.28 (−0.53,-0.04)			0.024	41.5
Ultradistal forearm bone mineral density	6	−0.17 (−0.57,0.23)			0.394	40.3
Lumbar spine bone mineral density	6	0.03 (−0.30,0.36)			0.868	40.3
Femoral neck bone mineral density	6	0.09 (−0.19,0.38)			0.518	40.3

#### 3.1.1 Age at menarche and BMD

We observed that an increase in AAM had a significant impact on BMD (β: 0.06, 95% CI: 0.10 to −0.02, *p*-value: 6.80E-03) and heel calcaneus BMD (β: 0.07, 95% CI: 0.11 to −0.02, *p*-value: 2.21E-03) when used as an exposure variable. However, this effect was not significant in FA-BMD, LS-BMD, and FN-BMD.

#### 3.1.2 Age at first live birth and BMD

When AFB was used as the exposure variable, we found a significant correlation between FA-BMD (β: 0.20, 95% CI: 0.37 to −0.02, *p*-value: 2.78E-02) and the exposure variable. This suggests a negative causal relationship between age at first live birth and FA-BMD.

#### 3.1.3 Age at menopause and BMD

When ANM was used as the exposure variable, an increase in menopausal age was significantly correlated with BMD (β: 0.05, 95% CI: 0.02 to 0.09, *p*-value: 2.91E-03), heel calcaneus BMD (β: 0.06, 95% CI: 0.02 to 0.09, *p*-value: 6.14E-04), FA-BMD (β: 0.10, 95% CI: 0.03 to 0.16, *p*-value: 2.53E-03), and LS-BMD (β: 0.10, 95% CI: 0.03 to 0.17, *p*-value: 4.91E-03). This suggests a positive causal relationship between menopausal age and BMD, heel calcaneus bone mineral density, ulnar bone density at the ultra-distal forearm, and lumbar spine bone mineral density.

#### 3.1.4 Age at last live birth and BMD

ALB was significantly correlated with BMD (β: 0.25, 95% CI: 0.49 to −0.01, *p*-value: 4.10E-02) and heel calcaneus BMD (β: 0.28, 95% CI: 0.53 to −0.04, *p*-value: 2.43E-02), showing a negative causal relationship.

### 3.2 Multivariable MR

**FIGURE 1:**
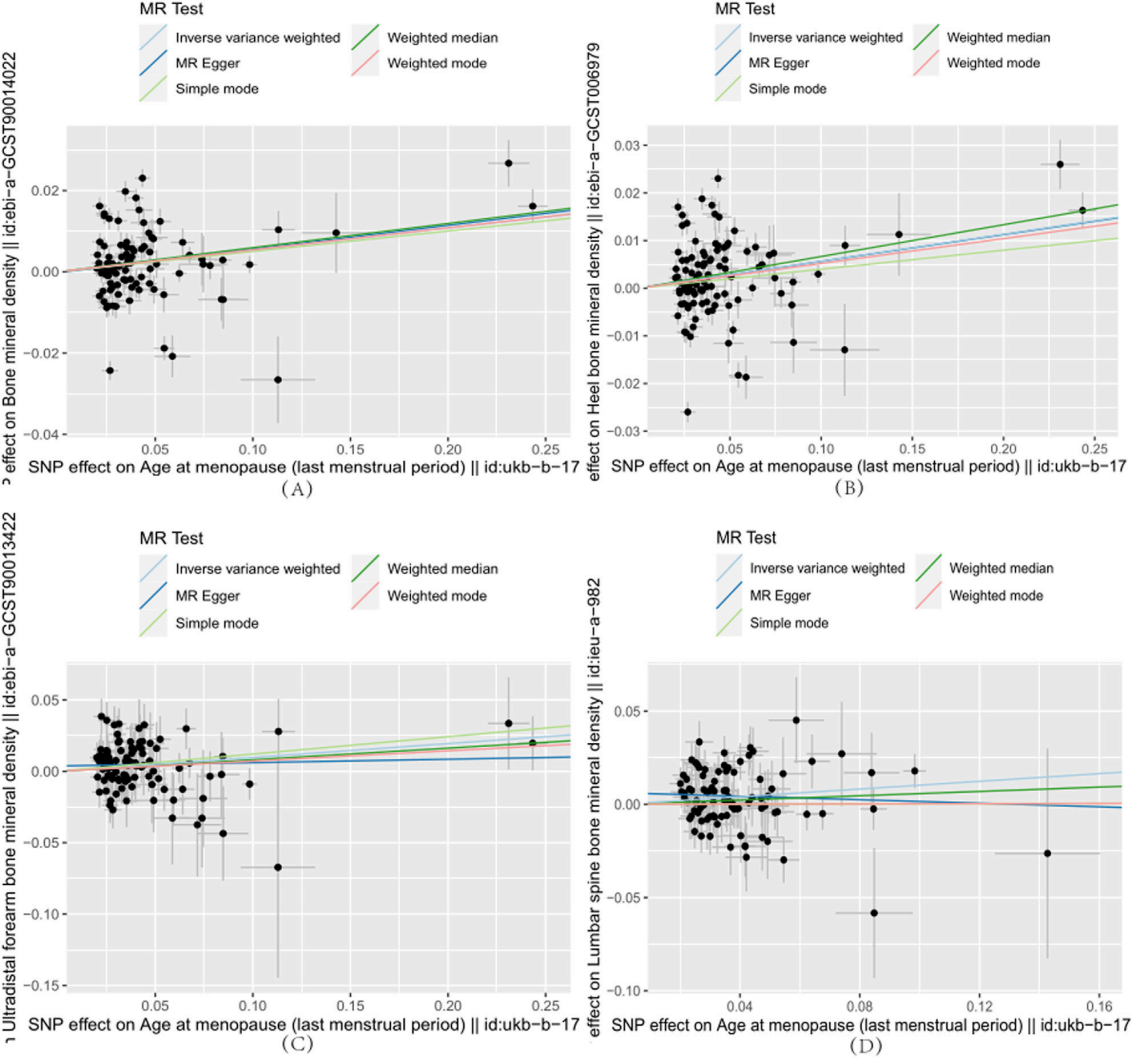
Each point on the plot represents an SNP locus. The *x*-axis represents the effect of SNP loci on the exposure variable, which corresponds to reproductive factors. The *y*-axis represents the effect of SNP loci on the outcome variable, which refers to the age at menopause. The lines in different colors represent the results of MR fitting. **(A)**–**(D)** indicate that age at menopause is positively associated with whole-body bone mineral density, heel bone mineral density, forearm ultradistal bone mineral density, and lumbar spine bone mineral density, respectively.

Furthermore, we employed a multivariable MR approach to analyze the simultaneous exposure to four reproductive factors as well as the BMD and three specific bone mineral density outcomes. The findings are presented in Table 2 and Figure 1, wherein the results are succinctly outlined.

**TABLE 2:** Multivariate Mendelian Randomization.

Exposure	snps	beta (95%Cl)	P
Bone mineral density			
Age at menarche	2	−0.02 (-0.06,0.02)	0.168
Age at first live birth	13	0.01 (-0.24,0.27)	0.161
Age at menopause	71	0.06 (0.03,0.09)	<0.001
Age at last live birth	1	−0.21 (-0.50,0.09)	0.055
Heel bone mineral density			
Age at menarche	4	−0.03 (-0.06,0.01)	0.101
Age at first live birth	13	−0.06 (0.16,0.29)	0.092
Age at menopause	82	0.06 (0.03,0.09)	<0.001
Age at last live birth	1	−0.40 (-0.78,-0.02)	0.037
Ultradistal forearm bone mineral density			
Age at menarche	4	−0.01 (-0.09,0.06)	0.600
Age at first live birth	14	−0.08 (-0.56,0.41)	0.436
Age at menopause	78	0.11 (0.05,0.18)	0.002
Age at last live birth	1	−0.17 (-0.74,0.4)	0.870
Lumbar spine bone mineral density			
Age at menarche	3	−0.03 (-0.1,0.05)	0.247
Age at first live birth	12	−0.1 (-0.57,0.37)	0.565
Age at menopause	73	0.08 (0.01,0.15)	0.003
Age at last live birth	1	−0.01 (-0.57,0.56)	0.553
Femoral neck bone mineral density			
Age at menarche	3	−0.06 (-0.12,0.01)	0.121
Age at first live birth	12	0.05 (-0.34,0.45)	0.399
Age at menopause	73	0.03 (-0.03,0.09)	0.076
Age at last live birth	1	−0.21 (-0.68,0.26)	0.473

In Table 2, we observed that after adjusting for AAM, AFB, and ALB, ANM remained significantly associated with BMD (β: 0.06, 95% CI:0.03 to 0.09, *p*-value: 2.54E-04), heel calcaneus BMD(β: 0.006, 95% CI:0.03 to 0.09, *p*-value: 1.00E-04), FA-BMD(β: 0.11, 95% CI:0.05 to 0.18, *p*-value: 1.77E-03), and LS-BMD(β: 0.08, 95% CI:0.01 to 0.15, *p*-value: 3.20E-03). This indicates a positive causal relationship between menopausal age and these bone density measures. Additionally, there was a negative causal relationship between age at last live birth and heel calcaneus BMD (β: 0.40, 95% CI: 0.78 to −0.02, *p*-value: 3.71E-02). However, there was no causal relationship between FN-BMD and any of the reproductive factors.

## 4 Discussion

We conducted a comprehensive MR analysis aiming to explore the causal relationship between reproductive factors in this study, such as menopausal age, and female bone density. The outcomes from the univariable MR analysis indicated a positive correlation between menopausal age and both BMD and bone density at various locations, suggesting that a delayed onset of menopause might confer a safeguarding effect on bone density. On the other hand, AAM, AFB, and ALB were negatively associated with bone density at different regions. In this study, considering the potential interaction effects among these four reproductive factors, we employed the multivariable MR analysis method for the first time. The results further confirmed the positive correlation between later menopausal age and BMD, and revealed for the first time a negative association between age at last live birth and heel calcaneus BMD. This study provides ample evidence that early menopausal age and late age at last live birth are risk factors for decreased female BMD.

Late AAM is believed to be associated with an increased risk of osteoporosis (Zhang et al., 2018; Yang et al., 2023), and our study further supports this finding. Additionally, estrogen levels are closely related to bone density (Eriksson et al., 2018), and female bone formation and growth are highly dependent on estrogen (Riggs et al., 2002). The metabolic effects of AAM on bone density may be influenced by the duration of estrogen exposure. This could be attributed to the shorter duration of estrogen exposure in women who experience a later age at menarche, thereby reducing the risk of developing osteoporosis. A cross-sectional study involving multiple exposure factors and bone density at various sites has shown that late age at menarche may affect peak bone mass in the lower lumbar spine of females, increasing their susceptibility to osteoporosis later in life (Chang et al., 2017). In contrast to these studies, we employed the MR study method, and our results not only demonstrated a strong correlation between Age at menarche and both BMD and heel calcaneus BMD but also validated the causal effect between them. However, this phenomenon was not observed in the results of the multivariable MR analysis, which may be due to the greater influence of other reproductive factors included in this study on the outcomes.

The mechanistic link between reproductive age and bone density remains unclear. Multiple studies have found a significant association between reproductive history and osteoporosis, but the research findings are controversial. For instance, a cross-sectional study suggested that adolescent pregnancy may not have a significant impact on bone mass acquisition and may not represent a risk factor for future osteoporosis (Miglioli et al., 2007). However, another cross-sectional study using KNHANES data demonstrated that teenage pregnancy could be a predictive indicator for postmenopausal osteoporosis in women (Cho et al., 2012). Previous studies primarily relied on cross-sectional designs or smaller cohort studies, and causal relationships have not been established.

In our study, for the first time, a univariate MR approach was utilized to establish a negative association between AFB and FA-BMD. Additionally, a negative correlation was observed between ALB and BMD. Thus, we established a causal relationship between reproductive age and BMD, which may be related to bone mass accumulation. During adolescence, bone mass accumulates rapidly, and there is some restorative capacity for bone loss. However, as peak bone mass is approached, the accumulation rate slows down. Pregnancy during this critical period can lead to bone loss, interrupting the crucial moment of bone mass accumulation (Yun et al., 2015), consequently resulting in decreased bone density. After reaching peak bone mass, bone loss begins around the age of 40 and accelerates during perimenopause. Women who give birth after the age of 35 may not recover bone density quickly during pregnancy (We et al., 2018). Therefore, women who become pregnant after the age of 35, with advanced maternal age during their last childbirth, may not fully recover their bone density and potentially increase the risk of postmenopausal osteoporosis. Additionally, women with a higher number of pregnancies usually have a relatively older age at their last childbirth, and multiparity also impacts bone density (Demir et al., 2008). Considering the potential interaction among these four reproductive factors, we adjusted for AAM, age at menopause, and age at first live birth in our analysis, conducting further multivariate MR studies. The results showed a significant attenuation in the association between age at last childbirth and BMD. However, it remained significantly associated with heel calcaneus BMD. Our findings unequivocally demonstrate that age at last childbirth is a risk factor for heel calcaneal bone density. This result provides important reference for clinical practice, reminding clinicians and women to pay attention to reproductive age planning and bone density assessment, aiming to prevent early the skeletal diseases related to osteoporosis and improve the health status of elderly women.

According to a longitudinal cohort study on menopausal transition (MT), the age at menopause has been found to be strongly correlated with postmenopausal bone density and may increase the susceptibility to menopausal osteoporotic fractures (Shieh et al., 2022). In our study, we found a positive correlation between the ANM and various measures of bone density, including BMD, heel calcaneus BMD, FA-BMD, LS-BMD. Furthermore, through a multivariable MR analysis that accounted for the influence of three other reproductive factors, we demonstrated that the association between the age at natural menopause and bone density remained significant, confirming it as an undeniable risk factor for bone density. In the aging process after menopause, the level of estrogen secreted by the ovaries gradually decreases. A molecular study on the basis of osteoporosis has found that estrogen and the WNT signaling pathway are closely related to its pathogenic mechanism (Wang et al., 2024). The WNT signaling pathway is involved in skeletal development and the maintenance of skeletal stability (Yang et al., 2020). Firstly, activation of the WNT signaling pathway can promote the proliferation and differentiation of stem cells into osteoblasts, thereby increasing bone density. Secondly, the WNT signaling pathway can also inhibit the activity of bone resorption cells, thereby maintaining the stability of bone quality and the healthy level of bone density (Baron and Kneissel, 2013). After menopause, the decline in estrogen levels leads to the inhibition of WNT signaling pathway activity, among which there is a correlation between Wnt16 and bone density (Koller et al., 2013). One fundamental research found that knockout of the Wnt16 gene in mice results in a decrease in bone density (Zheng et al., 2012). Also, the reduced estrogen levels increase the sensitivity of the bones to parathyroid hormone (PTH), resulting in a higher rate of bone breakdown compared to bone synthesis, ultimately causing osteoporosis (Cannata-Andía et al., 2010). Furthermore, the decline in estrogen levels also leads to dysregulation of various cytokines secretion in the body, such as disruptions in fatty acids and their metabolic pathways, which are important factors affecting bone density and may ultimately contribute to osteoporosis (Gong et al., 2021).

To the best of our knowledge, this study, which investigates the relationship between multiple reproductive factors as exposure variables and multi-site BMD as the outcome variable, is the first univariate and multivariate MR analysis of its kind. The strength of this study lies in its MR design, which eliminates the confounding effects and helps establish causal relationships. In addition, we combine univariate and multivariate MR to further understand the individual and combined effects of different variables on the experiment, obtaining more robust and powerful correlations by controlling for confounding biases. However, this study also has some limitations. On one hand, we only included genetic information from European populations in the GWAS database, and SNPs from different ethnicities may have different impacts on bone density. On the other hand, this study did not stratify the bone density SNPs by age, indicating the need for further research in this area.

## 5 Conclusion

In summary, this study convincingly demonstrates the causal relationship between multiple reproductive factors and BMD. Risk factors associated with decreased bone density were identified in the univariate MR analysis, including early onset of menopause, delayed onset of menarche, delayed age at first childbirth, and delayed age at last childbirth. Furthermore, in the multivariate MR analysis, the importance of early menopause age and late age at last childbirth in relation to decreased bone density was reaffirmed. The results of this study emphasize the importance of reproductive factors such as early menopause and late childbirth in osteoporosis screening and management, providing a new perspective on osteoporosis prevention in women. Therefore, it is recommended to incorporate reproductive factors into the existing osteoporosis management system to more comprehensively guide clinical practice, helping women better prevent and combat bone loss.

## Data Availability

The data presented in the study are deposited in the IEU database, Visit the website: https://gwas.mrcieu.ac.uk/, accession number ebi-a-GCST90014022 (Bone mineral density), ebi-a-GCST006979 (Heel bone mineral density), ebi-a-GCST90013422 (Ultradistal forearm bone mineral density), ieu-a-982 (Lumbar spine bone mineral density), ieu-a-980 (Femoral neck bone mineral density), ukb-b-12405 (Age at first live birth), ieu-b-4822 (Age at menarche), ukb-b-17422 (Age at menopause), ukb-b-8727 (Age at last live birth).
