# Supplementary material for: Causal relationship between reproductive factors and female bone density: a univariate and multivariate mendelian randomization study
Source: Front Genet. 2024 Sep 13;15:1393106. doi: 10.3389/fgene.2024.1393106 (PMC11427258; doi:10.3389/fgene.2024.1393106)
Supplement: Supplementary file 1 [file Table1.DOCX]

Supplementary 1

| exposure | Unit | participants included in analysis | GWAS ID | Year |
| --- | --- | --- | --- | --- |
| Age at first live birth | SD | 17049 EDI^[[1]](#footnote-0)^ | ukb-b-12405 | 2018 |
| Age at menarche | SD | 29346 EDI^1^ | ieu-b-4822 | 2022 |
| Age at menopause | SD | 143819 EDI^1^ | ukb-b-17422 | 2018 |
| Age at last live birth | SD | 170248 EDI^1^ | ukb-b-8727 | 2018 |

Supplementary 2

| outcome | Unit | participants included in analysis | GWAS ID | Year |
| --- | --- | --- | --- | --- |
| Bone mineral density | SD (g/cm^2) | 365403 EDI^1^ | ebi-a-GCST90014022 | 2021 |
| Heel bone mineral density | SD (g/cm^2) | 426824 EDI^1^ | ebi-a-GCST006979 | 2019 |
| Ultradistal forearm bone mineral density | SD (g/cm^2) | 21907 EDI^1^ | ebi-a-GCST90013422 | 2020 |
| Lumbar spine bone mineral density | SD (g/cm^2) | 28498 MDI^[[2]](#footnote-1)^ | ieu-a-982 | 2015 |
| Femoral neck bone mineral density | SD (g/cm^2) | 32735 MDI^2^ | ieu-a-980 | 2015 |

**Supplementary Table 3-22 shows the SNPs evaluating the causal associations between different reproductive factors and bone mineral density (BMD) in different sites. CHR represents chromosome number; SNP represents single nucleotide polymorphism; EA represents effect_allele; OA represents other_allele; SE represents standard error.**

Supplementary 3

| CHR | SNP | EA | OA | Age at first live birth | | |  | Bone mineral density | | |
| --- | --- | --- | --- | --- | --- | --- | --- | --- | --- | --- |
|  |  |  |  | β | SE | P |  | β | SE | P |
| 7 | rs10266297 | T | C | -1.90E-02 | 3.41E-03 | 2.60E-08 |  | 5.22E-03 | 2.12E-03 | 1.40E-02 |
| 2 | rs10496880 | C | T | -3.67E-02 | 6.66E-03 | 3.60E-08 |  | -4.53E-03 | 4.11E-03 | 2.70E-01 |
| 9 | rs10733289 | A | G | 1.93E-02 | 3.41E-03 | 1.50E-08 |  | -6.95E-04 | 2.09E-03 | 7.40E-01 |
| 7 | rs113905912 | C | T | 2.33E-02 | 3.52E-03 | 4.10E-11 |  | 2.33E-03 | 2.19E-03 | 2.88E-01 |
| 4 | rs11938781 | C | T | -2.66E-02 | 4.52E-03 | 4.10E-09 |  | 1.60E-02 | 2.79E-03 | 8.76E-09 |
| 1 | rs12089815 | A | G | 1.98E-02 | 3.37E-03 | 4.50E-09 |  | -3.84E-03 | 2.08E-03 | 6.47E-02 |
| 10 | rs12250380 | G | A | 2.18E-02 | 3.40E-03 | 1.50E-10 |  | 5.04E-04 | 2.09E-03 | 8.10E-01 |
| 14 | rs12435203 | A | G | -2.14E-02 | 3.69E-03 | 6.40E-09 |  | 5.81E-05 | 2.26E-03 | 9.79E-01 |
| 9 | rs12555870 | G | A | 1.91E-02 | 3.38E-03 | 1.70E-08 |  | 5.47E-03 | 2.07E-03 | 8.26E-03 |
| 12 | rs1401989 | T | C | -1.96E-02 | 3.53E-03 | 2.70E-08 |  | 3.11E-03 | 2.17E-03 | 1.51E-01 |
| 4 | rs17314832 | T | C | 2.01E-02 | 3.49E-03 | 7.80E-09 |  | -8.21E-04 | 2.14E-03 | 7.01E-01 |
| 18 | rs1941954 | C | A | 2.15E-02 | 3.57E-03 | 1.80E-09 |  | 4.57E-03 | 2.19E-03 | 3.68E-02 |
| 5 | rs2135029 | A | G | 1.90E-02 | 3.44E-03 | 3.10E-08 |  | 3.61E-03 | 2.11E-03 | 8.68E-02 |
| 12 | rs2456973 | C | A | 2.13E-02 | 3.52E-03 | 1.50E-09 |  | 8.91E-03 | 2.16E-03 | 3.76E-05 |
| 2 | rs2570497 | T | C | -2.28E-02 | 3.50E-03 | 8.00E-11 |  | 4.13E-04 | 2.16E-03 | 8.48E-01 |
| 12 | rs2645977 | A | G | 2.05E-02 | 3.48E-03 | 4.00E-09 |  | -8.00E-04 | 2.14E-03 | 7.09E-01 |
| 2 | rs2708146 | G | A | -2.04E-02 | 3.37E-03 | 1.60E-09 |  | -4.24E-03 | 2.09E-03 | 4.24E-02 |
| 16 | rs35489785 | G | C | 2.11E-02 | 3.81E-03 | 3.10E-08 |  | -2.31E-03 | 2.34E-03 | 3.24E-01 |
| 4 | rs362307 | T | C | -3.81E-02 | 6.42E-03 | 2.80E-09 |  | -6.28E-03 | 3.92E-03 | 1.09E-01 |
| 1 | rs3791129 | A | G | 2.18E-02 | 3.82E-03 | 1.10E-08 |  | -4.38E-03 | 2.37E-03 | 6.42E-02 |
| 6 | rs4305732 | G | A | 2.36E-02 | 3.48E-03 | 1.20E-11 |  | 9.84E-03 | 2.15E-03 | 4.88E-06 |
| 2 | rs4443016 | G | C | 1.89E-02 | 3.37E-03 | 1.90E-08 |  | 3.88E-03 | 2.08E-03 | 6.28E-02 |
| 7 | rs55773130 | C | T | 2.72E-02 | 4.33E-03 | 3.30E-10 |  | 2.49E-03 | 2.70E-03 | 3.56E-01 |
| 3 | rs6446187 | A | C | -3.42E-02 | 3.34E-03 | 1.30E-24 |  | 1.86E-02 | 2.05E-03 | 1.57E-19 |
| 14 | rs6574760 | C | A | -1.88E-02 | 3.41E-03 | 3.30E-08 |  | -1.03E-03 | 2.09E-03 | 6.22E-01 |
| 12 | rs704061 | C | T | -1.90E-02 | 3.36E-03 | 1.60E-08 |  | -3.01E-03 | 2.06E-03 | 1.45E-01 |
| 10 | rs7896518 | G | A | 1.94E-02 | 3.42E-03 | 1.40E-08 |  | -3.47E-03 | 2.10E-03 | 9.89E-02 |
| 19 | rs8110682 | C | T | 2.30E-02 | 3.54E-03 | 8.30E-11 |  | 1.87E-03 | 2.18E-03 | 3.91E-01 |
| 18 | rs896686 | G | T | 2.63E-02 | 4.50E-03 | 5.30E-09 |  | 1.45E-03 | 2.75E-03 | 5.98E-01 |
| 1 | rs909001 | G | C | -2.70E-02 | 4.44E-03 | 1.30E-09 |  | 4.23E-03 | 2.74E-03 | 1.22E-01 |
| 6 | rs9372625 | A | G | 2.40E-02 | 3.45E-03 | 3.60E-12 |  | 2.57E-04 | 2.14E-03 | 9.04E-01 |
| 2 | rs951807 | C | T | -2.51E-02 | 3.41E-03 | 1.70E-13 |  | 5.97E-03 | 2.11E-03 | 4.62E-03 |
| 3 | rs9882678 | T | G | -2.60E-02 | 4.40E-03 | 3.70E-09 |  | 2.11E-03 | 2.71E-03 | 4.36E-01 |
| 17 | rs9901404 | G | A | 2.00E-02 | 3.64E-03 | 4.10E-08 |  | -6.67E-03 | 2.23E-03 | 2.81E-03 |
| 21 | rs9980269 | A | G | 2.27E-02 | 4.12E-03 | 3.50E-08 |  | 3.80E-03 | 2.52E-03 | 1.32E-01 |

Supplementary 4

| CHR | SNP | EA | OA | Age at first live birth | | |  | Heel bone mineral density | | |
| --- | --- | --- | --- | --- | --- | --- | --- | --- | --- | --- |
|  |  |  |  | β | SE | P |  | β | SE | P |
| 7 | rs10266297 | T | C | -1.90E-02 | 3.41E-03 | 2.60E-08 |  | 6.19E-03 | 1.87E-03 | 2.20E-02 |
| 2 | rs10496880 | C | T | -3.67E-02 | 6.66E-03 | 3.60E-08 |  | -5.80E-03 | 3.64E-03 | 1.20E-01 |
| 9 | rs10733289 | A | G | 1.93E-02 | 3.41E-03 | 1.50E-08 |  | 9.45E-04 | 1.89E-03 | 8.30E-01 |
| 7 | rs113905912 | C | T | 2.33E-02 | 3.52E-03 | 4.10E-11 |  | -2.87E-04 | 1.93E-03 | 4.70E-01 |
| 4 | rs11938781 | C | T | -2.66E-02 | 4.52E-03 | 4.10E-09 |  | 1.60E-02 | 2.51E-03 | 1.70E-08 |
| 1 | rs12089815 | A | G | 1.98E-02 | 3.37E-03 | 4.50E-09 |  | -3.41E-03 | 1.84E-03 | 1.30E-01 |
| 10 | rs12250380 | G | A | 2.18E-02 | 3.40E-03 | 1.50E-10 |  | 1.87E-03 | 1.87E-03 | 2.60E-01 |
| 14 | rs12435203 | A | G | -2.14E-02 | 3.69E-03 | 6.40E-09 |  | 1.42E-03 | 2.04E-03 | 7.10E-01 |
| 9 | rs12555870 | G | A | 1.91E-02 | 3.38E-03 | 1.70E-08 |  | 5.49E-03 | 1.87E-03 | 2.30E-03 |
| 12 | rs1401989 | T | C | -1.96E-02 | 3.53E-03 | 2.70E-08 |  | 2.41E-03 | 1.95E-03 | 3.80E-01 |
| 4 | rs17314832 | T | C | 2.01E-02 | 3.49E-03 | 7.80E-09 |  | -1.78E-03 | 1.92E-03 | 5.90E-01 |
| 18 | rs1941954 | C | A | 2.15E-02 | 3.57E-03 | 1.80E-09 |  | 4.90E-03 | 1.98E-03 | 1.20E-02 |
| 5 | rs2135029 | A | G | 1.90E-02 | 3.44E-03 | 3.10E-08 |  | 4.57E-03 | 1.90E-03 | 4.60E-02 |
| 12 | rs2456973 | C | A | 2.13E-02 | 3.52E-03 | 1.50E-09 |  | 9.71E-03 | 1.94E-03 | 2.20E-06 |
| 2 | rs2570497 | T | C | -2.28E-02 | 3.50E-03 | 8.00E-11 |  | 1.98E-03 | 1.91E-03 | 3.80E-01 |
| 12 | rs2645977 | A | G | 2.05E-02 | 3.48E-03 | 4.00E-09 |  | 1.70E-03 | 1.95E-03 | 3.10E-01 |
| 2 | rs2708146 | G | A | -2.04E-02 | 3.37E-03 | 1.60E-09 |  | -2.15E-03 | 1.85E-03 | 9.60E-02 |
| 16 | rs35489785 | G | C | 2.11E-02 | 3.81E-03 | 3.10E-08 |  | -1.89E-03 | 2.11E-03 | 4.10E-01 |
| 4 | rs362307 | T | C | -3.81E-02 | 6.42E-03 | 2.80E-09 |  | -5.35E-03 | 3.52E-03 | 1.40E-01 |
| 1 | rs3791129 | A | G | 2.18E-02 | 3.82E-03 | 1.10E-08 |  | -5.31E-03 | 2.10E-03 | 1.60E-02 |
| 6 | rs4305732 | G | A | 2.36E-02 | 3.48E-03 | 1.20E-11 |  | 9.07E-03 | 1.90E-03 | 2.00E-04 |
| 2 | rs4443016 | G | C | 1.89E-02 | 3.37E-03 | 1.90E-08 |  | 3.99E-03 | 1.84E-03 | 4.80E-03 |
| 7 | rs55773130 | C | T | 2.72E-02 | 4.33E-03 | 3.30E-10 |  | 6.53E-03 | 2.37E-03 | 6.80E-03 |
| 3 | rs6446187 | A | C | -3.42E-02 | 3.34E-03 | 1.30E-24 |  | 1.77E-02 | 1.85E-03 | 1.50E-19 |
| 14 | rs6574760 | C | A | -1.88E-02 | 3.41E-03 | 3.30E-08 |  | -1.27E-03 | 1.89E-03 | 1.70E-01 |
| 12 | rs704061 | C | T | -1.90E-02 | 3.36E-03 | 1.60E-08 |  | -5.34E-03 | 1.86E-03 | 5.40E-03 |
| 10 | rs7896518 | G | A | 1.94E-02 | 3.42E-03 | 1.40E-08 |  | -2.91E-03 | 1.85E-03 | 4.20E-01 |
| 18 | rs896686 | G | T | 2.63E-02 | 4.50E-03 | 5.30E-09 |  | 3.41E-03 | 2.49E-03 | 2.80E-01 |
| 1 | rs909001 | G | C | -2.70E-02 | 4.44E-03 | 1.30E-09 |  | 2.17E-03 | 2.43E-03 | 1.20E-01 |
| 6 | rs9372625 | A | G | 2.40E-02 | 3.45E-03 | 3.60E-12 |  | -1.76E-04 | 1.89E-03 | 8.90E-01 |
| 2 | rs951807 | C | T | -2.51E-02 | 3.41E-03 | 1.70E-13 |  | 4.55E-03 | 1.87E-03 | 1.90E-02 |
| 3 | rs9882678 | T | G | -2.60E-02 | 4.40E-03 | 3.70E-09 |  | 1.26E-03 | 2.43E-03 | 7.30E-01 |
| 21 | rs9980269 | A | G | 2.27E-02 | 4.12E-03 | 3.50E-08 |  | 2.32E-03 | 2.29E-03 | 5.90E-01 |

Supplementary 5

| CHR | SNP | EA | OA | Age at first live birth | | |  | Ultradistal forearm bone mineral density | | |
| --- | --- | --- | --- | --- | --- | --- | --- | --- | --- | --- |
|  |  |  |  | β | SE | P |  | β | SE | P |
| 7 | rs10266297 | T | C | -1.90E-02 | 3.41E-03 | 2.60E-08 |  | -6.09E-03 | 1.10E-02 | 5.78E-01 |
| 2 | rs10496880 | C | T | -3.67E-02 | 6.66E-03 | 3.60E-08 |  | 1.97E-02 | 2.02E-02 | 3.29E-01 |
| 9 | rs10733289 | A | G | 1.93E-02 | 3.41E-03 | 1.50E-08 |  | 5.71E-03 | 1.09E-02 | 6.01E-01 |
| 7 | rs113905912 | C | T | 2.33E-02 | 3.52E-03 | 4.10E-11 |  | -7.98E-03 | 1.12E-02 | 4.76E-01 |
| 4 | rs11938781 | C | T | -2.66E-02 | 4.52E-03 | 4.10E-09 |  | 9.24E-03 | 1.49E-02 | 5.34E-01 |
| 1 | rs12089815 | A | G | 1.98E-02 | 3.37E-03 | 4.50E-09 |  | -1.10E-02 | 1.07E-02 | 3.03E-01 |
| 10 | rs12250380 | G | A | 2.18E-02 | 3.40E-03 | 1.50E-10 |  | -1.46E-02 | 1.09E-02 | 1.80E-01 |
| 14 | rs12435203 | A | G | -2.14E-02 | 3.69E-03 | 6.40E-09 |  | -6.24E-03 | 1.17E-02 | 5.96E-01 |
| 9 | rs12555870 | G | A | 1.91E-02 | 3.38E-03 | 1.70E-08 |  | 7.07E-05 | 1.07E-02 | 9.95E-01 |
| 12 | rs1401989 | T | C | -1.96E-02 | 3.53E-03 | 2.70E-08 |  | 1.48E-02 | 1.10E-02 | 1.78E-01 |
| 4 | rs17314832 | T | C | 2.01E-02 | 3.49E-03 | 7.80E-09 |  | -1.26E-02 | 1.11E-02 | 2.54E-01 |
| 18 | rs1941954 | C | A | 2.15E-02 | 3.57E-03 | 1.80E-09 |  | 1.34E-02 | 1.14E-02 | 2.43E-01 |
| 5 | rs2135029 | A | G | 1.90E-02 | 3.44E-03 | 3.10E-08 |  | -4.57E-03 | 1.08E-02 | 6.71E-01 |
| 12 | rs2456973 | C | A | 2.13E-02 | 3.52E-03 | 1.50E-09 |  | -1.43E-02 | 1.13E-02 | 2.07E-01 |
| 2 | rs2570497 | T | C | -2.28E-02 | 3.50E-03 | 8.00E-11 |  | 1.53E-02 | 1.10E-02 | 1.62E-01 |
| 12 | rs2645977 | A | G | 2.05E-02 | 3.48E-03 | 4.00E-09 |  | -2.78E-03 | 1.10E-02 | 8.01E-01 |
| 2 | rs2708146 | G | A | -2.04E-02 | 3.37E-03 | 1.60E-09 |  | 2.08E-02 | 1.06E-02 | 5.07E-02 |
| 16 | rs35489785 | G | C | 2.11E-02 | 3.81E-03 | 3.10E-08 |  | -5.22E-03 | 1.22E-02 | 6.67E-01 |
| 4 | rs362307 | T | C | -3.81E-02 | 6.42E-03 | 2.80E-09 |  | 9.92E-03 | 1.95E-02 | 6.12E-01 |
| 1 | rs3791129 | A | G | 2.18E-02 | 3.82E-03 | 1.10E-08 |  | -4.13E-03 | 1.26E-02 | 7.44E-01 |
| 6 | rs4305732 | G | A | 2.36E-02 | 3.48E-03 | 1.20E-11 |  | 1.06E-02 | 1.11E-02 | 3.41E-01 |
| 2 | rs4443016 | G | C | 1.89E-02 | 3.37E-03 | 1.90E-08 |  | 2.03E-03 | 1.06E-02 | 8.49E-01 |
| 7 | rs55773130 | C | T | 2.72E-02 | 4.33E-03 | 3.30E-10 |  | -6.84E-03 | 1.37E-02 | 6.19E-01 |
| 3 | rs6446187 | A | C | -3.42E-02 | 3.34E-03 | 1.30E-24 |  | 4.54E-03 | 1.06E-02 | 6.69E-01 |
| 14 | rs6574760 | C | A | -1.88E-02 | 3.41E-03 | 3.30E-08 |  | 1.45E-03 | 1.08E-02 | 8.93E-01 |
| 12 | rs704061 | C | T | -1.90E-02 | 3.36E-03 | 1.60E-08 |  | -2.32E-02 | 1.09E-02 | 3.32E-02 |
| 10 | rs7896518 | G | A | 1.94E-02 | 3.42E-03 | 1.40E-08 |  | -8.82E-03 | 1.08E-02 | 4.12E-01 |
| 18 | rs896686 | G | T | 2.63E-02 | 4.50E-03 | 5.30E-09 |  | -1.31E-02 | 1.46E-02 | 3.68E-01 |
| 1 | rs909001 | G | C | -2.70E-02 | 4.44E-03 | 1.30E-09 |  | 1.69E-02 | 1.37E-02 | 2.17E-01 |
| 6 | rs9372625 | A | G | 2.40E-02 | 3.45E-03 | 3.60E-12 |  | 1.71E-02 | 1.09E-02 | 1.17E-01 |
| 2 | rs951807 | C | T | -2.51E-02 | 3.41E-03 | 1.70E-13 |  | 7.19E-03 | 1.10E-02 | 5.12E-01 |
| 3 | rs9882678 | T | G | -2.60E-02 | 4.40E-03 | 3.70E-09 |  | 1.81E-02 | 1.43E-02 | 2.06E-01 |
| 17 | rs9901404 | G | A | 2.00E-02 | 3.64E-03 | 4.10E-08 |  | -1.18E-02 | 1.13E-02 | 3.00E-01 |
| 21 | rs9980269 | A | G | 2.27E-02 | 4.12E-03 | 3.50E-08 |  | 7.31E-03 | 1.27E-02 | 5.66E-01 |

Supplementary 6

| CHR | SNP | EA | OA | Age at first live birth | | |  | Lumbar spine bone mineral density | | |
| --- | --- | --- | --- | --- | --- | --- | --- | --- | --- | --- |
|  |  |  |  | β | SE | P |  | β | SE | P |
| 7 | rs10266297 | T | C | -1.90E-02 | 3.41E-03 | 2.60E-08 |  | 7.06E-03 | 8.93E-03 | 4.40E-01 |
| 2 | rs10496880 | C | T | -3.67E-02 | 6.66E-03 | 3.60E-08 |  | 3.95E-03 | 1.75E-02 | 8.26E-01 |
| 9 | rs10733289 | A | G | 1.93E-02 | 3.41E-03 | 1.50E-08 |  | 8.39E-03 | 8.90E-03 | 3.58E-01 |
| 7 | rs113905912 | C | T | 2.33E-02 | 3.52E-03 | 4.10E-11 |  | -3.53E-03 | 9.08E-03 | 7.05E-01 |
| 4 | rs11938781 | C | T | -2.66E-02 | 4.52E-03 | 4.10E-09 |  | -1.88E-02 | 1.19E-02 | 1.22E-01 |
| 1 | rs12089815 | A | G | 1.98E-02 | 3.37E-03 | 4.50E-09 |  | -2.68E-03 | 8.78E-03 | 7.66E-01 |
| 10 | rs12250380 | G | A | 2.18E-02 | 3.40E-03 | 1.50E-10 |  | 1.47E-03 | 8.86E-03 | 8.71E-01 |
| 14 | rs12435203 | A | G | -2.14E-02 | 3.69E-03 | 6.40E-09 |  | 5.02E-03 | 9.60E-03 | 6.09E-01 |
| 9 | rs12555870 | G | A | 1.91E-02 | 3.38E-03 | 1.70E-08 |  | -4.51E-03 | 8.84E-03 | 6.18E-01 |
| 12 | rs1401989 | T | C | -1.96E-02 | 3.53E-03 | 2.70E-08 |  | -2.02E-03 | 9.24E-03 | 8.31E-01 |
| 4 | rs17314832 | T | C | 2.01E-02 | 3.49E-03 | 7.80E-09 |  | 1.09E-02 | 9.05E-03 | 2.41E-01 |
| 18 | rs1941954 | C | A | 2.15E-02 | 3.57E-03 | 1.80E-09 |  | 1.49E-02 | 9.34E-03 | 1.19E-01 |
| 5 | rs2135029 | A | G | 1.90E-02 | 3.44E-03 | 3.10E-08 |  | -6.33E-03 | 9.06E-03 | 4.95E-01 |
| 12 | rs2456973 | C | A | 2.13E-02 | 3.52E-03 | 1.50E-09 |  | -8.59E-03 | 9.36E-03 | 3.70E-01 |
| 2 | rs2570497 | T | C | -2.28E-02 | 3.50E-03 | 8.00E-11 |  | -2.36E-03 | 9.09E-03 | 8.00E-01 |
| 12 | rs2645977 | A | G | 2.05E-02 | 3.48E-03 | 4.00E-09 |  | 9.19E-03 | 9.18E-03 | 3.28E-01 |
| 2 | rs2708146 | G | A | -2.04E-02 | 3.37E-03 | 1.60E-09 |  | 1.17E-04 | 8.76E-03 | 9.90E-01 |
| 16 | rs35489785 | G | C | 2.11E-02 | 3.81E-03 | 3.10E-08 |  | -1.26E-02 | 9.99E-03 | 2.19E-01 |
| 1 | rs3791129 | A | G | 2.18E-02 | 3.82E-03 | 1.10E-08 |  | 6.85E-03 | 1.02E-02 | 5.10E-01 |
| 6 | rs4305732 | G | A | 2.36E-02 | 3.48E-03 | 1.20E-11 |  | -5.46E-03 | 9.22E-03 | 5.63E-01 |
| 2 | rs4443016 | G | C | 1.89E-02 | 3.37E-03 | 1.90E-08 |  | -5.33E-03 | 8.87E-03 | 5.57E-01 |
| 7 | rs55773130 | C | T | 2.72E-02 | 4.33E-03 | 3.30E-10 |  | -1.69E-04 | 1.10E-02 | 9.88E-01 |
| 3 | rs6446187 | A | C | -3.42E-02 | 3.34E-03 | 1.30E-24 |  | -5.90E-05 | 8.94E-03 | 9.95E-01 |
| 14 | rs6574760 | C | A | -1.88E-02 | 3.41E-03 | 3.30E-08 |  | 6.60E-03 | 8.86E-03 | 4.67E-01 |
| 12 | rs704061 | C | T | -1.90E-02 | 3.36E-03 | 1.60E-08 |  | 7.16E-03 | 8.71E-03 | 4.22E-01 |
| 10 | rs7896518 | G | A | 1.94E-02 | 3.42E-03 | 1.40E-08 |  | -7.20E-05 | 8.81E-03 | 9.94E-01 |
| 19 | rs8110682 | C | T | 2.30E-02 | 3.54E-03 | 8.30E-11 |  | 1.35E-02 | 1.02E-02 | 1.96E-01 |
| 18 | rs896686 | G | T | 2.63E-02 | 4.50E-03 | 5.30E-09 |  | 2.74E-02 | 1.15E-02 | 2.04E-02 |
| 1 | rs909001 | G | C | -2.70E-02 | 4.44E-03 | 1.30E-09 |  | 3.18E-04 | 1.15E-02 | 9.79E-01 |
| 6 | rs9372625 | A | G | 2.40E-02 | 3.45E-03 | 3.60E-12 |  | 6.29E-03 | 9.07E-03 | 4.98E-01 |
| 2 | rs951807 | C | T | -2.51E-02 | 3.41E-03 | 1.70E-13 |  | -9.65E-03 | 8.90E-03 | 2.90E-01 |
| 3 | rs9882678 | T | G | -2.60E-02 | 4.40E-03 | 3.70E-09 |  | -5.87E-03 | 1.14E-02 | 6.15E-01 |
| 21 | rs9980269 | A | G | 2.27E-02 | 4.12E-03 | 3.50E-08 |  | 4.75E-03 | 1.08E-02 | 6.68E-01 |

Supplementary 7

| CHR | SNP | EA | OA | Age at first live birth | | |  | Femoral neck bone mineral density | | |
| --- | --- | --- | --- | --- | --- | --- | --- | --- | --- | --- |
|  |  |  |  | β | SE | P |  | β | SE | P |
| 7 | rs10266297 | T | C | -1.90E-02 | 3.41E-03 | 2.60E-08 |  | 1.30E-02 | 7.63E-03 | 9.53E-02 |
| 2 | rs10496880 | C | T | -3.67E-02 | 6.66E-03 | 3.60E-08 |  | -2.27E-03 | 1.51E-02 | 8.83E-01 |
| 9 | rs10733289 | A | G | 1.93E-02 | 3.41E-03 | 1.50E-08 |  | 3.39E-03 | 7.65E-03 | 6.64E-01 |
| 7 | rs113905912 | C | T | 2.33E-02 | 3.52E-03 | 4.10E-11 |  | 5.28E-03 | 7.82E-03 | 5.08E-01 |
| 4 | rs11938781 | C | T | -2.66E-02 | 4.52E-03 | 4.10E-09 |  | 1.87E-03 | 1.02E-02 | 8.58E-01 |
| 1 | rs12089815 | A | G | 1.98E-02 | 3.37E-03 | 4.50E-09 |  | 3.75E-04 | 7.55E-03 | 9.61E-01 |
| 10 | rs12250380 | G | A | 2.18E-02 | 3.40E-03 | 1.50E-10 |  | 6.16E-03 | 7.61E-03 | 4.29E-01 |
| 14 | rs12435203 | A | G | -2.14E-02 | 3.69E-03 | 6.40E-09 |  | 1.32E-02 | 8.28E-03 | 1.18E-01 |
| 9 | rs12555870 | G | A | 1.91E-02 | 3.38E-03 | 1.70E-08 |  | -2.66E-03 | 7.58E-03 | 7.31E-01 |
| 12 | rs1401989 | T | C | -1.96E-02 | 3.53E-03 | 2.70E-08 |  | -5.95E-03 | 7.95E-03 | 4.64E-01 |
| 4 | rs17314832 | T | C | 2.01E-02 | 3.49E-03 | 7.80E-09 |  | 2.03E-02 | 7.78E-03 | 1.07E-02 |
| 18 | rs1941954 | C | A | 2.15E-02 | 3.57E-03 | 1.80E-09 |  | 7.13E-04 | 8.00E-03 | 9.31E-01 |
| 5 | rs2135029 | A | G | 1.90E-02 | 3.44E-03 | 3.10E-08 |  | -8.81E-03 | 7.77E-03 | 2.67E-01 |
| 12 | rs2456973 | C | A | 2.13E-02 | 3.52E-03 | 1.50E-09 |  | -5.45E-03 | 8.04E-03 | 5.07E-01 |
| 2 | rs2570497 | T | C | -2.28E-02 | 3.50E-03 | 8.00E-11 |  | 1.50E-02 | 7.81E-03 | 6.10E-02 |
| 12 | rs2645977 | A | G | 2.05E-02 | 3.48E-03 | 4.00E-09 |  | 9.22E-03 | 7.87E-03 | 2.52E-01 |
| 2 | rs2708146 | G | A | -2.04E-02 | 3.37E-03 | 1.60E-09 |  | -4.49E-03 | 7.53E-03 | 5.60E-01 |
| 16 | rs35489785 | G | C | 2.11E-02 | 3.81E-03 | 3.10E-08 |  | -1.37E-02 | 8.58E-03 | 1.18E-01 |
| 1 | rs3791129 | A | G | 2.18E-02 | 3.82E-03 | 1.10E-08 |  | 1.32E-02 | 8.74E-03 | 1.39E-01 |
| 6 | rs4305732 | G | A | 2.36E-02 | 3.48E-03 | 1.20E-11 |  | 6.23E-03 | 7.89E-03 | 4.40E-01 |
| 2 | rs4443016 | G | C | 1.89E-02 | 3.37E-03 | 1.90E-08 |  | -5.40E-03 | 7.59E-03 | 4.86E-01 |
| 7 | rs55773130 | C | T | 2.72E-02 | 4.33E-03 | 3.30E-10 |  | 6.04E-04 | 9.43E-03 | 9.50E-01 |
| 3 | rs6446187 | A | C | -3.42E-02 | 3.34E-03 | 1.30E-24 |  | -5.42E-03 | 7.64E-03 | 4.88E-01 |
| 14 | rs6574760 | C | A | -1.88E-02 | 3.41E-03 | 3.30E-08 |  | 3.35E-03 | 7.61E-03 | 6.67E-01 |
| 12 | rs704061 | C | T | -1.90E-02 | 3.36E-03 | 1.60E-08 |  | -5.47E-03 | 7.49E-03 | 4.74E-01 |
| 10 | rs7896518 | G | A | 1.94E-02 | 3.42E-03 | 1.40E-08 |  | -1.23E-02 | 7.54E-03 | 1.11E-01 |
| 19 | rs8110682 | C | T | 2.30E-02 | 3.54E-03 | 8.30E-11 |  | 2.20E-03 | 8.72E-03 | 8.05E-01 |
| 18 | rs896686 | G | T | 2.63E-02 | 4.50E-03 | 5.30E-09 |  | 7.84E-03 | 9.90E-03 | 4.38E-01 |
| 1 | rs909001 | G | C | -2.70E-02 | 4.44E-03 | 1.30E-09 |  | 4.56E-03 | 9.98E-03 | 6.55E-01 |
| 6 | rs9372625 | A | G | 2.40E-02 | 3.45E-03 | 3.60E-12 |  | 5.15E-03 | 7.79E-03 | 5.17E-01 |
| 2 | rs951807 | C | T | -2.51E-02 | 3.41E-03 | 1.70E-13 |  | -9.62E-04 | 7.63E-03 | 9.02E-01 |
| 3 | rs9882678 | T | G | -2.60E-02 | 4.40E-03 | 3.70E-09 |  | -8.30E-03 | 9.82E-03 | 4.08E-01 |
| 21 | rs9980269 | A | G | 2.27E-02 | 4.12E-03 | 3.50E-08 |  | 7.16E-03 | 9.32E-03 | 4.52E-01 |

Supplementary 8

| CHR | SNP | EA | OA | Age at menarche | | |  | Bone mineral density | | |
| --- | --- | --- | --- | --- | --- | --- | --- | --- | --- | --- |
|  |  |  |  | β | SE | P |  | β | SE | P |
| 9 | rs10978435 | C | T | -8.98E-02 | 1.44E-02 | 4.13E-10 |  | 2.29E-04 | 2.21E-03 | 9.17E-01 |
| 9 | rs16924631 | C | G | -9.04E-02 | 1.64E-02 | 3.67E-08 |  | -3.45E-03 | 2.97E-03 | 2.45E-01 |
| 9 | rs2090409 | A | C | -9.67E-02 | 1.30E-02 | 1.05E-13 |  | 4.17E-04 | 2.21E-03 | 8.50E-01 |
| 16 | rs2362643 | A | G | -8.74E-02 | 1.34E-02 | 7.39E-11 |  | 2.96E-03 | 2.18E-03 | 1.76E-01 |
| 6 | rs314268 | A | G | -1.40E-01 | 1.28E-02 | 5.09E-28 |  | 1.33E-02 | 2.19E-03 | 1.38E-09 |
| 1 | rs543874 | G | A | -7.92E-02 | 1.44E-02 | 3.80E-08 |  | 1.28E-02 | 2.54E-03 | 4.73E-07 |
| 11 | rs7114175 | T | A | 7.46E-02 | 1.28E-02 | 6.26E-09 |  | -9.58E-03 | 2.07E-03 | 3.74E-06 |
| 2 | rs72887143 | T | A | 8.79E-02 | 1.59E-02 | 3.55E-08 |  | -1.37E-03 | 2.78E-03 | 6.23E-01 |
| 1 | rs79627842 | C | T | -1.30E-01 | 1.82E-02 | 8.23E-13 |  | 6.29E-03 | 3.08E-03 | 4.09E-02 |

Supplementary 9

| CHR | SNP | EA | OA | Age at menarche | | |  | Heel bone mineral density | | |
| --- | --- | --- | --- | --- | --- | --- | --- | --- | --- | --- |
|  |  |  |  | β | SE | P |  | β | SE | P |
| 9 | rs16924631 | C | G | -9.04E-02 | 1.64E-02 | 3.67E-08 |  | -4.96E-03 | 2.68E-03 | 2.60E-02 |
| 9 | rs2090409 | A | C | -9.67E-02 | 1.30E-02 | 1.05E-13 |  | 1.42E-03 | 2.00E-03 | 9.90E-01 |
| 16 | rs2362643 | A | G | -8.74E-02 | 1.34E-02 | 7.39E-11 |  | 1.50E-03 | 1.99E-03 | 9.40E-01 |
| 6 | rs314268 | A | G | -1.40E-01 | 1.28E-02 | 5.09E-28 |  | 1.37E-02 | 1.94E-03 | 9.10E-10 |
| 1 | rs543874 | G | A | -7.92E-02 | 1.44E-02 | 3.80E-08 |  | 1.18E-02 | 2.26E-03 | 3.60E-06 |
| 11 | rs7114175 | T | A | 7.46E-02 | 1.28E-02 | 6.26E-09 |  | -8.92E-03 | 1.85E-03 | 9.30E-07 |
| 2 | rs72887143 | T | A | 8.79E-02 | 1.59E-02 | 3.55E-08 |  | -2.53E-03 | 2.46E-03 | 4.60E-01 |
| 1 | rs79627842 | C | T | -1.30E-01 | 1.82E-02 | 8.23E-13 |  | 6.36E-03 | 2.74E-03 | 6.80E-02 |

Supplementary 10

| CHR | SNP | EA | OA | Age at menarche | | |  | Ultradistal forearm bone mineral density | | |
| --- | --- | --- | --- | --- | --- | --- | --- | --- | --- | --- |
|  |  |  |  | β | SE | P |  | β | SE | P |
| 9 | rs10978435 | C | T | -8.98E-02 | 1.44E-02 | 4.13E-10 |  | -9.38E-03 | 1.12E-02 | 4.03E-01 |
| 9 | rs16924631 | C | G | -9.04E-02 | 1.64E-02 | 3.67E-08 |  | 5.96E-03 | 1.40E-02 | 6.69E-01 |
| 9 | rs2090409 | A | C | -9.67E-02 | 1.30E-02 | 1.05E-13 |  | -8.43E-03 | 1.12E-02 | 4.53E-01 |
| 16 | rs2362643 | A | G | -8.74E-02 | 1.34E-02 | 7.39E-11 |  | -6.78E-03 | 1.14E-02 | 5.52E-01 |
| 6 | rs314268 | A | G | -1.40E-01 | 1.28E-02 | 5.09E-28 |  | -4.31E-03 | 1.09E-02 | 6.94E-01 |
| 1 | rs543874 | G | A | -7.92E-02 | 1.44E-02 | 3.80E-08 |  | 2.95E-03 | 1.24E-02 | 8.11E-01 |
| 11 | rs7114175 | T | A | 7.46E-02 | 1.28E-02 | 6.26E-09 |  | -1.60E-03 | 1.07E-02 | 8.82E-01 |
| 2 | rs72887143 | T | A | 8.79E-02 | 1.59E-02 | 3.55E-08 |  | -4.23E-03 | 1.45E-02 | 7.70E-01 |
| 1 | rs79627842 | C | T | -1.30E-01 | 1.82E-02 | 8.23E-13 |  | -1.27E-02 | 1.60E-02 | 4.25E-01 |

Supplementary 11

| CHR | SNP | EA | OA | Age at menarche | | |  | Lumbar spine bone mineral density | | |
| --- | --- | --- | --- | --- | --- | --- | --- | --- | --- | --- |
|  |  |  |  | β | SE | P |  | β | SE | P |
| 9 | rs16924631 | C | G | -9.04E-02 | 1.64E-02 | 3.67E-08 |  | 1.30E-02 | 1.23E-02 | 3.02E-01 |
| 9 | rs2090409 | A | C | -9.67E-02 | 1.30E-02 | 1.05E-13 |  | -4.75E-03 | 9.34E-03 | 6.19E-01 |
| 16 | rs2362643 | A | G | -8.74E-02 | 1.34E-02 | 7.39E-11 |  | 3.78E-03 | 9.24E-03 | 6.89E-01 |
| 6 | rs314268 | A | G | -1.40E-01 | 1.28E-02 | 5.09E-28 |  | 1.54E-02 | 9.17E-03 | 9.99E-02 |
| 1 | rs543874 | G | A | -7.92E-02 | 1.44E-02 | 3.80E-08 |  | 6.67E-03 | 1.11E-02 | 5.57E-01 |
| 11 | rs7114175 | T | A | 7.46E-02 | 1.28E-02 | 6.26E-09 |  | -2.74E-02 | 8.73E-03 | 2.18E-03 |
| 2 | rs72887143 | T | A | 8.79E-02 | 1.59E-02 | 3.55E-08 |  | -1.71E-02 | 1.17E-02 | 1.52E-01 |

Supplementary 12

| CHR | SNP | EA | OA | Age at menarche | | |  | Femoral neck bone mineral density | | |
| --- | --- | --- | --- | --- | --- | --- | --- | --- | --- | --- |
|  |  |  |  | β | SE | P |  | β | SE | P |
| 9 | rs16924631 | C | G | -9.04E-02 | 1.64E-02 | 3.67E-08 |  | 2.08E-02 | 1.06E-02 | 5.47E-02 |
| 9 | rs2090409 | A | C | -9.67E-02 | 1.30E-02 | 1.05E-13 |  | 6.95E-03 | 8.04E-03 | 3.98E-01 |
| 16 | rs2362643 | A | G | -8.74E-02 | 1.34E-02 | 7.39E-11 |  | -1.03E-02 | 7.92E-03 | 2.03E-01 |
| 6 | rs314268 | A | G | -1.40E-01 | 1.28E-02 | 5.09E-28 |  | 1.82E-02 | 7.89E-03 | 2.40E-02 |
| 1 | rs543874 | G | A | -7.92E-02 | 1.44E-02 | 3.80E-08 |  | 2.42E-03 | 9.49E-03 | 8.03E-01 |
| 11 | rs7114175 | T | A | 7.46E-02 | 1.28E-02 | 6.26E-09 |  | -1.30E-02 | 7.49E-03 | 8.92E-02 |
| 2 | rs72887143 | T | A | 8.79E-02 | 1.59E-02 | 3.55E-08 |  | -1.50E-02 | 1.01E-02 | 1.47E-01 |

Supplementary 13

| CHR | SNP | EA | OA | Age at menopause | | |  | Bone mineral density | | |
| --- | --- | --- | --- | --- | --- | --- | --- | --- | --- | --- |
|  |  |  |  | β | SE | P |  | β | SE | P |
| 11 | rs1020622 | G | C | 2.19E-02 | 3.73E-03 | 4.70E-09 |  | -9.19E-04 | 2.09E-03 | 6.59E-01 |
| 5 | rs10476835 | A | G | 2.11E-02 | 3.69E-03 | 1.00E-08 |  | 2.17E-03 | 2.06E-03 | 2.91E-01 |
| 16 | rs10521305 | C | T | 5.44E-02 | 7.78E-03 | 2.70E-12 |  | -5.64E-03 | 4.33E-03 | 1.93E-01 |
| 9 | rs10813912 | G | A | 2.49E-02 | 3.80E-03 | 6.00E-11 |  | -7.81E-04 | 2.12E-03 | 7.13E-01 |
| 20 | rs10854167 | C | G | -3.95E-02 | 4.51E-03 | 1.90E-18 |  | -7.52E-03 | 2.51E-03 | 2.70E-03 |
| 3 | rs10937153 | A | G | 3.61E-02 | 4.33E-03 | 7.40E-17 |  | 2.95E-03 | 2.40E-03 | 2.20E-01 |
| 10 | rs10998203 | G | C | -3.61E-02 | 4.58E-03 | 3.30E-15 |  | -5.59E-03 | 2.56E-03 | 2.91E-02 |
| 11 | rs11031005 | C | T | 5.46E-02 | 5.27E-03 | 3.90E-25 |  | -1.88E-02 | 2.95E-03 | 1.75E-10 |
| 7 | rs112190116 | T | C | 1.43E-01 | 1.76E-02 | 5.10E-16 |  | 9.60E-03 | 9.86E-03 | 3.30E-01 |
| 15 | rs112217463 | A | G | -3.73E-02 | 3.82E-03 | 1.50E-22 |  | 1.82E-03 | 2.13E-03 | 3.93E-01 |
| 13 | rs11571818 | C | T | -1.13E-01 | 1.91E-02 | 3.30E-09 |  | 2.66E-02 | 1.07E-02 | 1.27E-02 |
| 17 | rs11650324 | G | A | 3.91E-02 | 4.43E-03 | 1.10E-18 |  | 5.15E-03 | 2.47E-03 | 3.70E-02 |
| 1 | rs12046563 | G | A | -2.52E-02 | 4.32E-03 | 5.50E-09 |  | -1.53E-04 | 2.42E-03 | 9.50E-01 |
| 1 | rs12132692 | T | C | 4.03E-02 | 6.27E-03 | 1.30E-10 |  | -2.86E-03 | 3.51E-03 | 4.17E-01 |
| 16 | rs12444283 | G | C | -2.89E-02 | 3.96E-03 | 2.70E-13 |  | -2.82E-03 | 2.20E-03 | 2.00E-01 |
| 4 | rs12503643 | T | G | 4.67E-02 | 3.77E-03 | 2.60E-35 |  | 9.55E-03 | 2.10E-03 | 5.49E-06 |
| 19 | rs12609254 | T | C | -3.39E-02 | 5.95E-03 | 1.20E-08 |  | 1.90E-03 | 3.31E-03 | 5.66E-01 |
| 4 | rs156520 | A | C | 2.25E-02 | 4.12E-03 | 4.90E-08 |  | -3.52E-04 | 2.29E-03 | 8.78E-01 |
| 4 | rs1565909 | T | C | 6.23E-02 | 3.69E-03 | 3.50E-64 |  | -3.94E-04 | 2.05E-03 | 8.48E-01 |
| 6 | rs1655907 | C | T | -2.90E-02 | 5.09E-03 | 1.10E-08 |  | -1.06E-03 | 2.85E-03 | 7.11E-01 |
| 20 | rs16991615 | A | G | 2.43E-01 | 7.66E-03 | 1.00E-200 |  | 1.62E-02 | 4.17E-03 | 1.05E-04 |
| 14 | rs1760940 | C | A | -3.81E-02 | 4.26E-03 | 4.40E-19 |  | 3.83E-03 | 2.38E-03 | 1.07E-01 |
| 3 | rs17646517 | G | C | -7.16E-02 | 1.23E-02 | 5.10E-09 |  | -7.89E-03 | 6.93E-03 | 2.54E-01 |
| 17 | rs17650301 | C | A | -4.01E-02 | 4.04E-03 | 2.80E-23 |  | -1.82E-02 | 2.25E-03 | 7.09E-16 |
| 16 | rs17680522 | G | A | 2.32E-02 | 4.06E-03 | 1.20E-08 |  | 4.76E-04 | 2.25E-03 | 8.33E-01 |
| 2 | rs17820747 | C | A | 2.60E-02 | 4.34E-03 | 2.10E-09 |  | -8.46E-03 | 2.43E-03 | 5.01E-04 |
| 14 | rs17856037 | T | C | -7.40E-02 | 1.17E-02 | 2.90E-10 |  | -3.09E-03 | 6.49E-03 | 6.34E-01 |
| 12 | rs1790123 | T | C | 3.47E-02 | 4.60E-03 | 4.70E-14 |  | 1.98E-02 | 2.59E-03 | 2.20E-14 |
| 12 | rs184540366 | T | G | -8.48E-02 | 1.29E-02 | 4.60E-11 |  | 6.85E-03 | 7.20E-03 | 3.42E-01 |
| 1 | rs200448 | C | T | -2.15E-02 | 3.73E-03 | 8.10E-09 |  | -1.62E-02 | 2.09E-03 | 8.32E-15 |
| 15 | rs2241522 | G | A | -2.16E-02 | 3.90E-03 | 2.90E-08 |  | -7.36E-03 | 2.17E-03 | 6.77E-04 |
| 12 | rs2277339 | G | T | -7.81E-02 | 6.00E-03 | 9.70E-39 |  | -1.54E-03 | 3.38E-03 | 6.48E-01 |
| 19 | rs2304192 | G | A | -2.82E-02 | 4.04E-03 | 3.00E-12 |  | -1.75E-03 | 2.25E-03 | 4.38E-01 |
| 16 | rs251723 | C | G | 5.17E-02 | 3.88E-03 | 2.00E-40 |  | -8.97E-03 | 2.16E-03 | 3.37E-05 |
| 7 | rs2519673 | A | G | -2.68E-02 | 3.82E-03 | 2.40E-12 |  | -1.35E-04 | 2.15E-03 | 9.50E-01 |
| 6 | rs2524119 | C | T | 2.38E-02 | 3.69E-03 | 1.20E-10 |  | -7.33E-03 | 2.08E-03 | 4.14E-04 |
| 3 | rs2624847 | T | G | 2.68E-02 | 4.22E-03 | 2.10E-10 |  | -2.43E-02 | 2.35E-03 | 3.58E-25 |
| 5 | rs2688194 | C | T | 4.41E-02 | 6.66E-03 | 3.60E-11 |  | 1.21E-02 | 3.70E-03 | 1.09E-03 |
| 12 | rs28416520 | A | G | -3.56E-02 | 3.73E-03 | 1.20E-21 |  | -2.05E-03 | 2.08E-03 | 3.24E-01 |
| 6 | rs2844466 | C | T | -4.72E-02 | 3.84E-03 | 8.30E-35 |  | -4.87E-03 | 2.16E-03 | 2.39E-02 |
| 8 | rs28797500 | C | T | -8.46E-02 | 4.43E-03 | 3.80E-81 |  | -2.90E-03 | 2.45E-03 | 2.38E-01 |
| 17 | rs2941506 | G | A | 3.74E-02 | 3.97E-03 | 5.20E-21 |  | 2.20E-03 | 2.21E-03 | 3.21E-01 |
| 19 | rs299168 | A | G | 5.06E-02 | 6.29E-03 | 8.00E-16 |  | 1.90E-03 | 3.48E-03 | 5.85E-01 |
| 3 | rs345985 | T | C | -2.39E-02 | 3.75E-03 | 1.90E-10 |  | -1.38E-02 | 2.09E-03 | 3.70E-11 |
| 4 | rs34811474 | A | G | -2.39E-02 | 4.37E-03 | 4.50E-08 |  | -1.43E-02 | 2.43E-03 | 3.79E-09 |
| 19 | rs349306 | A | G | 5.25E-02 | 5.61E-03 | 8.60E-21 |  | 1.24E-02 | 3.15E-03 | 8.12E-05 |
| 19 | rs34962991 | A | G | -9.84E-02 | 3.83E-03 | 2.90E-145 |  | -1.77E-03 | 2.14E-03 | 4.07E-01 |
| 17 | rs3803756 | T | A | -2.48E-02 | 3.84E-03 | 1.20E-10 |  | 1.14E-03 | 2.14E-03 | 5.94E-01 |
| 19 | rs394448 | C | G | -2.05E-02 | 3.70E-03 | 3.20E-08 |  | 2.57E-03 | 2.05E-03 | 2.11E-01 |
| 7 | rs4049337 | C | G | -2.57E-02 | 4.02E-03 | 1.50E-10 |  | -3.26E-03 | 2.26E-03 | 1.50E-01 |
| 5 | rs419128 | A | G | -3.48E-02 | 3.86E-03 | 2.20E-19 |  | -3.82E-04 | 2.16E-03 | 8.59E-01 |
| 1 | rs4408133 | C | G | 3.16E-02 | 3.91E-03 | 6.90E-16 |  | -9.44E-05 | 2.19E-03 | 9.66E-01 |
| 2 | rs4491723 | G | A | 3.11E-02 | 4.18E-03 | 1.10E-13 |  | -5.57E-03 | 2.35E-03 | 1.78E-02 |
| 1 | rs4495657 | C | T | 3.28E-02 | 3.76E-03 | 2.40E-18 |  | 3.67E-03 | 2.10E-03 | 8.09E-02 |
| 2 | rs4668354 | G | C | 3.48E-02 | 3.80E-03 | 5.60E-20 |  | -1.20E-03 | 2.14E-03 | 5.74E-01 |
| 3 | rs4679121 | T | C | -3.81E-02 | 6.05E-03 | 3.10E-10 |  | -5.74E-03 | 3.36E-03 | 8.77E-02 |
| 6 | rs4716056 | G | A | 2.14E-02 | 3.79E-03 | 1.60E-08 |  | -5.98E-03 | 2.14E-03 | 5.18E-03 |
| 16 | rs4782369 | C | G | 3.00E-02 | 3.93E-03 | 2.10E-14 |  | -2.00E-03 | 2.19E-03 | 3.61E-01 |
| 22 | rs4821798 | C | T | -4.35E-02 | 3.99E-03 | 1.20E-27 |  | -2.31E-02 | 2.22E-03 | 2.83E-25 |
| 13 | rs4886238 | A | G | 3.80E-02 | 3.95E-03 | 6.30E-22 |  | 3.69E-03 | 2.19E-03 | 9.17E-02 |
| 3 | rs507926 | C | T | 2.99E-02 | 4.94E-03 | 1.50E-09 |  | -4.27E-04 | 2.76E-03 | 8.77E-01 |
| 20 | rs536092 | T | C | 2.38E-02 | 3.81E-03 | 3.90E-10 |  | 6.32E-03 | 2.12E-03 | 2.84E-03 |
| 1 | rs55707872 | C | A | 3.54E-02 | 3.98E-03 | 5.70E-19 |  | 7.31E-03 | 2.22E-03 | 9.92E-04 |
| 15 | rs55848327 | A | G | 2.52E-02 | 4.37E-03 | 8.80E-09 |  | -8.81E-03 | 2.44E-03 | 3.12E-04 |
| 5 | rs58279426 | C | T | 6.76E-02 | 3.70E-03 | 1.50E-74 |  | 4.09E-03 | 2.06E-03 | 4.71E-02 |
| 20 | rs6011452 | A | C | -4.91E-02 | 4.45E-03 | 2.80E-28 |  | -8.10E-03 | 2.50E-03 | 1.18E-03 |
| 19 | rs60907808 | G | A | -4.44E-02 | 5.26E-03 | 3.20E-17 |  | -6.24E-03 | 2.94E-03 | 3.37E-02 |
| 20 | rs6139074 | C | A | -3.06E-02 | 4.56E-03 | 1.80E-11 |  | 3.23E-03 | 2.54E-03 | 2.03E-01 |
| 10 | rs61870304 | G | A | -3.68E-02 | 6.11E-03 | 1.70E-09 |  | 7.11E-03 | 3.41E-03 | 3.69E-02 |
| 2 | rs62156695 | G | A | -6.41E-02 | 6.04E-03 | 2.70E-26 |  | -7.26E-03 | 3.40E-03 | 3.30E-02 |
| 7 | rs62445870 | T | C | 7.46E-02 | 1.24E-02 | 1.90E-09 |  | 1.86E-03 | 6.95E-03 | 7.90E-01 |
| 2 | rs6435156 | T | C | -2.63E-02 | 4.22E-03 | 4.50E-10 |  | -3.64E-03 | 2.37E-03 | 1.25E-01 |
| 8 | rs6470643 | C | A | -2.83E-02 | 4.47E-03 | 2.40E-10 |  | 8.36E-03 | 2.49E-03 | 7.67E-04 |
| 16 | rs6500437 | C | T | -3.55E-02 | 3.97E-03 | 4.60E-19 |  | -6.01E-03 | 2.21E-03 | 6.61E-03 |
| 10 | rs6584351 | G | A | 2.05E-02 | 3.68E-03 | 2.60E-08 |  | 4.17E-03 | 2.06E-03 | 4.25E-02 |
| 1 | rs6667957 | C | T | -2.60E-02 | 3.75E-03 | 4.30E-12 |  | 9.42E-04 | 2.09E-03 | 6.53E-01 |
| 2 | rs6736096 | C | T | 2.01E-02 | 3.68E-03 | 4.70E-08 |  | 8.46E-04 | 2.07E-03 | 6.82E-01 |
| 4 | rs6830848 | T | G | -3.48E-02 | 3.68E-03 | 3.60E-21 |  | 2.10E-03 | 2.06E-03 | 3.07E-01 |
| 6 | rs6912979 | C | T | -2.55E-02 | 4.09E-03 | 4.30E-10 |  | -2.08E-03 | 2.30E-03 | 3.65E-01 |
| 7 | rs6961014 | G | C | -2.90E-02 | 4.51E-03 | 1.20E-10 |  | -6.50E-03 | 2.54E-03 | 1.05E-02 |
| 10 | rs7087644 | G | A | -8.39E-02 | 9.31E-03 | 2.00E-19 |  | 6.78E-03 | 5.28E-03 | 1.99E-01 |
| 11 | rs7125555 | T | C | -2.76E-02 | 3.69E-03 | 6.50E-14 |  | -1.94E-03 | 2.06E-03 | 3.46E-01 |
| 1 | rs72708144 | C | T | 5.88E-02 | 9.21E-03 | 1.70E-10 |  | -2.08E-02 | 5.17E-03 | 5.84E-05 |
| 5 | rs72814771 | G | T | 4.94E-02 | 6.25E-03 | 2.90E-15 |  | -4.33E-03 | 3.47E-03 | 2.12E-01 |
| 2 | rs72827480 | C | T | 2.71E-02 | 3.76E-03 | 6.00E-13 |  | -4.48E-03 | 2.11E-03 | 3.36E-02 |
| 10 | rs728900 | A | T | -3.17E-02 | 3.76E-03 | 3.20E-17 |  | -7.08E-03 | 2.09E-03 | 7.18E-04 |
| 19 | rs73037453 | T | C | -2.64E-02 | 4.69E-03 | 1.70E-08 |  | -1.31E-02 | 2.60E-03 | 4.99E-07 |
| 20 | rs746748 | T | C | 4.74E-02 | 7.29E-03 | 7.50E-11 |  | 7.32E-04 | 4.03E-03 | 8.56E-01 |
| 10 | rs74701710 | A | G | -4.83E-02 | 8.04E-03 | 1.80E-09 |  | -8.45E-03 | 4.55E-03 | 6.32E-02 |
| 7 | rs74742883 | G | T | -3.11E-02 | 4.45E-03 | 3.00E-12 |  | -1.26E-02 | 2.52E-03 | 6.07E-07 |
| 12 | rs75770066 | G | A | 2.31E-01 | 1.06E-02 | 8.09E-105 |  | 2.67E-02 | 5.74E-03 | 3.18E-06 |
| 2 | rs7589040 | T | C | -3.15E-02 | 4.50E-03 | 2.40E-12 |  | -1.56E-03 | 2.57E-03 | 5.43E-01 |
| 4 | rs7661090 | T | C | -3.53E-02 | 5.96E-03 | 3.20E-09 |  | -4.39E-03 | 3.33E-03 | 1.88E-01 |
| 2 | rs76928871 | G | A | 4.30E-02 | 4.69E-03 | 4.30E-20 |  | 5.61E-03 | 2.62E-03 | 3.24E-02 |
| 12 | rs77100210 | C | A | 1.13E-01 | 8.44E-03 | 5.60E-41 |  | 1.04E-02 | 4.63E-03 | 2.52E-02 |
| 7 | rs7778113 | T | G | 2.80E-02 | 3.92E-03 | 9.40E-13 |  | 1.98E-03 | 2.21E-03 | 3.71E-01 |
| 1 | rs7779 | C | G | 3.99E-02 | 7.07E-03 | 1.70E-08 |  | 8.09E-04 | 3.96E-03 | 8.38E-01 |
| 8 | rs77952879 | C | G | -4.21E-02 | 6.95E-03 | 1.30E-09 |  | -8.84E-03 | 3.91E-03 | 2.36E-02 |
| 6 | rs78080415 | C | T | 2.98E-02 | 5.25E-03 | 1.30E-08 |  | -8.52E-03 | 2.96E-03 | 3.96E-03 |
| 8 | rs7827991 | A | C | 4.17E-02 | 6.88E-03 | 1.30E-09 |  | 1.52E-02 | 3.82E-03 | 6.74E-05 |
| 2 | rs78385274 | C | G | -5.94E-02 | 6.00E-03 | 4.60E-23 |  | -4.99E-03 | 3.39E-03 | 1.40E-01 |
| 2 | rs78385274 | C | G | -5.94E-02 | 6.00E-03 | 4.60E-23 |  | -7.51E-03 | 3.38E-03 | 2.62E-02 |
| 8 | rs7845046 | A | T | -4.92E-02 | 8.38E-03 | 4.40E-09 |  | 1.24E-02 | 4.69E-03 | 8.17E-03 |
| 16 | rs8045589 | T | A | -2.26E-02 | 3.70E-03 | 9.70E-10 |  | -3.19E-03 | 2.06E-03 | 1.21E-01 |
| 2 | rs809673 | G | A | -4.17E-02 | 3.77E-03 | 1.70E-28 |  | 4.46E-03 | 2.11E-03 | 3.44E-02 |
| 15 | rs888368 | G | A | 2.22E-02 | 3.68E-03 | 1.70E-09 |  | 7.77E-04 | 2.05E-03 | 7.05E-01 |
| 4 | rs9307242 | C | T | -2.72E-02 | 3.82E-03 | 1.20E-12 |  | 2.81E-03 | 2.14E-03 | 1.89E-01 |
| 5 | rs9313736 | A | G | -3.14E-02 | 3.77E-03 | 8.00E-17 |  | -1.32E-03 | 2.11E-03 | 5.30E-01 |
| 6 | rs9358956 | C | G | -6.59E-02 | 4.94E-03 | 1.60E-40 |  | -3.99E-03 | 2.75E-03 | 1.47E-01 |
| 1 | rs9438982 | A | C | -4.71E-02 | 3.95E-03 | 7.40E-33 |  | 2.89E-03 | 2.21E-03 | 1.90E-01 |
| 22 | rs9607474 | T | C | -3.24E-02 | 5.73E-03 | 1.50E-08 |  | -6.57E-03 | 3.19E-03 | 3.95E-02 |
| 22 | rs9613667 | C | A | -2.32E-02 | 3.88E-03 | 2.00E-09 |  | 4.76E-03 | 2.16E-03 | 2.75E-02 |
| 15 | rs9788714 | A | G | -4.29E-02 | 3.81E-03 | 1.80E-29 |  | 1.27E-03 | 2.12E-03 | 5.49E-01 |
| 3 | rs9818740 | A | G | -2.78E-02 | 4.14E-03 | 1.70E-11 |  | 3.88E-03 | 2.30E-03 | 9.20E-02 |
| 17 | rs9915489 | T | A | 4.36E-02 | 3.91E-03 | 7.20E-29 |  | 8.66E-03 | 2.18E-03 | 7.17E-05 |

Supplementary 14

| CHR | SNP | EA | OA | Age at menopause | | |  | Heel bone mineral density | | |
| --- | --- | --- | --- | --- | --- | --- | --- | --- | --- | --- |
|  |  |  |  | β | SE | P |  | β | SE | P |
| 11 | rs1020622 | G | C | 2.19E-02 | 3.73E-03 | 4.70E-09 |  | -2.71E-03 | 1.86E-03 | 2.30E-01 |
| 5 | rs10476835 | A | G | 2.11E-02 | 3.69E-03 | 1.00E-08 |  | 2.40E-03 | 1.85E-03 | 1.60E-01 |
| 16 | rs10521305 | C | T | 5.44E-02 | 7.78E-03 | 2.70E-12 |  | -2.45E-03 | 3.91E-03 | 3.90E-01 |
| 9 | rs10813912 | G | A | 2.49E-02 | 3.80E-03 | 6.00E-11 |  | -3.25E-03 | 1.91E-03 | 7.50E-02 |
| 20 | rs10854167 | C | G | -3.95E-02 | 4.51E-03 | 1.90E-18 |  | -6.09E-03 | 2.26E-03 | 1.20E-02 |
| 3 | rs10937153 | A | G | 3.61E-02 | 4.33E-03 | 7.40E-17 |  | 5.22E-03 | 2.16E-03 | 6.70E-02 |
| 10 | rs10998203 | G | C | -3.61E-02 | 4.58E-03 | 3.30E-15 |  | -6.86E-03 | 2.29E-03 | 3.60E-02 |
| 11 | rs11031005 | C | T | 5.46E-02 | 5.27E-03 | 3.90E-25 |  | -1.83E-02 | 2.63E-03 | 5.70E-12 |
| 7 | rs112190116 | T | C | 1.43E-01 | 1.76E-02 | 5.10E-16 |  | 1.13E-02 | 8.66E-03 | 3.40E-01 |
| 15 | rs112217463 | A | G | -3.73E-02 | 3.82E-03 | 1.50E-22 |  | 2.87E-03 | 1.93E-03 | 3.10E-01 |
| 13 | rs11571818 | C | T | -1.13E-01 | 1.91E-02 | 3.30E-09 |  | 1.29E-02 | 9.66E-03 | 4.90E-01 |
| 17 | rs11650324 | G | A | 3.91E-02 | 4.43E-03 | 1.10E-18 |  | 5.82E-03 | 2.22E-03 | 2.00E-02 |
| 1 | rs12046563 | G | A | -2.52E-02 | 4.32E-03 | 5.50E-09 |  | -8.68E-05 | 2.15E-03 | 4.70E-01 |
| 1 | rs12132692 | T | C | 4.03E-02 | 6.27E-03 | 1.30E-10 |  | -4.68E-03 | 3.11E-03 | 2.50E-02 |
| 4 | rs12503643 | T | G | 4.67E-02 | 3.77E-03 | 2.60E-35 |  | 8.52E-03 | 1.89E-03 | 3.00E-05 |
| 19 | rs12609254 | T | C | -3.39E-02 | 5.95E-03 | 1.20E-08 |  | 3.45E-04 | 3.00E-03 | 7.50E-01 |
| 4 | rs156520 | A | C | 2.25E-02 | 4.12E-03 | 4.90E-08 |  | -1.04E-03 | 2.06E-03 | 8.90E-02 |
| 4 | rs1565909 | T | C | 6.23E-02 | 3.69E-03 | 3.50E-64 |  | 6.71E-05 | 1.85E-03 | 8.10E-01 |
| 6 | rs1655907 | C | T | -2.90E-02 | 5.09E-03 | 1.10E-08 |  | -3.21E-03 | 2.52E-03 | 3.40E-01 |
| 20 | rs16991615 | A | G | 2.43E-01 | 7.66E-03 | 1.00E-200 |  | 1.63E-02 | 3.76E-03 | 7.80E-05 |
| 14 | rs1760940 | C | A | -3.81E-02 | 4.26E-03 | 4.40E-19 |  | 4.89E-03 | 2.15E-03 | 6.10E-02 |
| 3 | rs17646517 | G | C | -7.16E-02 | 1.23E-02 | 5.10E-09 |  | -6.95E-03 | 6.21E-03 | 2.00E-01 |
| 17 | rs17650301 | C | A | -4.01E-02 | 4.04E-03 | 2.80E-23 |  | -1.74E-02 | 2.03E-03 | 6.30E-18 |
| 16 | rs17680522 | G | A | 2.32E-02 | 4.06E-03 | 1.20E-08 |  | 1.14E-03 | 2.03E-03 | 3.70E-01 |
| 2 | rs17820747 | C | A | 2.60E-02 | 4.34E-03 | 2.10E-09 |  | -9.36E-03 | 2.15E-03 | 2.60E-04 |
| 14 | rs17856037 | T | C | -7.40E-02 | 1.17E-02 | 2.90E-10 |  | -7.38E-03 | 5.90E-03 | 5.00E-02 |
| 12 | rs1790123 | T | C | 3.47E-02 | 4.60E-03 | 4.70E-14 |  | 1.88E-02 | 2.33E-03 | 3.10E-11 |
| 12 | rs184540366 | T | G | -8.48E-02 | 1.29E-02 | 4.60E-11 |  | 1.14E-02 | 6.49E-03 | 1.70E-01 |
| 1 | rs200448 | C | T | -2.15E-02 | 3.73E-03 | 8.10E-09 |  | -1.70E-02 | 1.85E-03 | 2.40E-16 |
| 15 | rs2241522 | G | A | -2.16E-02 | 3.90E-03 | 2.90E-08 |  | -7.58E-03 | 1.96E-03 | 1.50E-04 |
| 12 | rs2277339 | G | T | -7.81E-02 | 6.00E-03 | 9.70E-39 |  | 1.09E-03 | 3.03E-03 | 5.10E-01 |
| 16 | rs251723 | C | G | 5.17E-02 | 3.88E-03 | 2.00E-40 |  | -8.78E-03 | 1.95E-03 | 2.50E-04 |
| 7 | rs2519673 | A | G | -2.68E-02 | 3.82E-03 | 2.40E-12 |  | 8.16E-04 | 1.89E-03 | 9.50E-01 |
| 3 | rs2624847 | T | G | 2.68E-02 | 4.22E-03 | 2.10E-10 |  | -2.60E-02 | 2.17E-03 | 6.10E-28 |
| 5 | rs2688194 | C | T | 4.41E-02 | 6.66E-03 | 3.60E-11 |  | 1.49E-02 | 3.33E-03 | 6.20E-06 |
| 12 | rs28416520 | A | G | -3.56E-02 | 3.73E-03 | 1.20E-21 |  | -1.39E-03 | 1.87E-03 | 2.50E-01 |
| 6 | rs2844466 | C | T | -4.72E-02 | 3.84E-03 | 8.30E-35 |  | -5.98E-03 | 1.91E-03 | 4.90E-03 |
| 8 | rs28797500 | C | T | -8.46E-02 | 4.43E-03 | 3.80E-81 |  | -1.29E-03 | 2.22E-03 | 6.30E-01 |
| 17 | rs2941506 | G | A | 3.74E-02 | 3.97E-03 | 5.20E-21 |  | 3.19E-04 | 1.99E-03 | 8.00E-01 |
| 19 | rs299168 | A | G | 5.06E-02 | 6.29E-03 | 8.00E-16 |  | 2.36E-03 | 3.15E-03 | 3.10E-01 |
| 3 | rs345985 | T | C | -2.39E-02 | 3.75E-03 | 1.90E-10 |  | -1.53E-02 | 1.88E-03 | 6.30E-14 |
| 4 | rs34811474 | A | G | -2.39E-02 | 4.37E-03 | 4.50E-08 |  | -1.32E-02 | 2.19E-03 | 9.40E-11 |
| 19 | rs349306 | A | G | 5.25E-02 | 5.61E-03 | 8.60E-21 |  | 1.21E-02 | 2.83E-03 | 1.50E-03 |
| 19 | rs34962991 | A | G | -9.84E-02 | 3.83E-03 | 2.90E-145 |  | -2.96E-03 | 1.93E-03 | 8.50E-02 |
| 17 | rs3803756 | T | A | -2.48E-02 | 3.84E-03 | 1.20E-10 |  | 3.18E-04 | 1.92E-03 | 4.40E-01 |
| 19 | rs394448 | C | G | -2.05E-02 | 3.70E-03 | 3.20E-08 |  | 9.64E-04 | 1.86E-03 | 4.70E-01 |
| 7 | rs4049337 | C | G | -2.57E-02 | 4.02E-03 | 1.50E-10 |  | -3.79E-03 | 1.99E-03 | 2.50E-01 |
| 5 | rs419128 | A | G | -3.48E-02 | 3.86E-03 | 2.20E-19 |  | 5.89E-04 | 1.94E-03 | 7.80E-01 |
| 1 | rs4408133 | C | G | 3.16E-02 | 3.91E-03 | 6.90E-16 |  | 2.25E-03 | 1.94E-03 | 3.50E-01 |
| 2 | rs4491723 | G | A | 3.11E-02 | 4.18E-03 | 1.10E-13 |  | -6.52E-03 | 2.08E-03 | 8.00E-03 |
| 1 | rs4495657 | C | T | 3.28E-02 | 3.76E-03 | 2.40E-18 |  | 2.90E-03 | 1.87E-03 | 8.50E-02 |
| 2 | rs4668354 | G | C | 3.48E-02 | 3.80E-03 | 5.60E-20 |  | -3.25E-03 | 1.89E-03 | 1.20E-01 |
| 3 | rs4679121 | T | C | -3.81E-02 | 6.05E-03 | 3.10E-10 |  | -2.83E-03 | 3.02E-03 | 1.60E-01 |
| 6 | rs4716056 | G | A | 2.14E-02 | 3.79E-03 | 1.60E-08 |  | -5.80E-03 | 1.89E-03 | 2.90E-03 |
| 16 | rs4782369 | C | G | 3.00E-02 | 3.93E-03 | 2.10E-14 |  | -3.15E-03 | 1.97E-03 | 6.40E-02 |
| 22 | rs4821798 | C | T | -4.35E-02 | 3.99E-03 | 1.20E-27 |  | -2.30E-02 | 2.01E-03 | 2.00E-22 |
| 13 | rs4886238 | A | G | 3.80E-02 | 3.95E-03 | 6.30E-22 |  | 2.38E-03 | 1.98E-03 | 2.00E-01 |
| 3 | rs507926 | C | T | 2.99E-02 | 4.94E-03 | 1.50E-09 |  | -6.17E-04 | 2.48E-03 | 9.10E-01 |
| 20 | rs536092 | T | C | 2.38E-02 | 3.81E-03 | 3.90E-10 |  | 5.83E-03 | 1.91E-03 | 1.10E-02 |
| 1 | rs55707872 | C | A | 3.54E-02 | 3.98E-03 | 5.70E-19 |  | 7.56E-03 | 1.97E-03 | 4.30E-04 |
| 15 | rs55848327 | A | G | 2.52E-02 | 4.37E-03 | 8.80E-09 |  | -9.16E-03 | 2.21E-03 | 2.50E-04 |
| 5 | rs58279426 | C | T | 6.76E-02 | 3.70E-03 | 1.50E-74 |  | 4.96E-03 | 1.85E-03 | 1.40E-02 |
| 20 | rs6011452 | A | C | -4.91E-02 | 4.45E-03 | 2.80E-28 |  | -9.45E-03 | 2.25E-03 | 2.40E-04 |
| 19 | rs60907808 | G | A | -4.44E-02 | 5.26E-03 | 3.20E-17 |  | -4.64E-03 | 2.66E-03 | 2.10E-01 |
| 20 | rs6139074 | C | A | -3.06E-02 | 4.56E-03 | 1.80E-11 |  | 1.23E-03 | 2.30E-03 | 9.80E-01 |
| 2 | rs62156695 | G | A | -6.41E-02 | 6.04E-03 | 2.70E-26 |  | -8.64E-03 | 3.01E-03 | 1.30E-02 |
| 7 | rs62445870 | T | C | 7.46E-02 | 1.24E-02 | 1.90E-09 |  | 2.16E-03 | 6.11E-03 | 9.40E-01 |
| 2 | rs6435156 | T | C | -2.63E-02 | 4.22E-03 | 4.50E-10 |  | -5.90E-03 | 2.10E-03 | 2.90E-02 |
| 8 | rs6470643 | C | A | -2.83E-02 | 4.47E-03 | 2.40E-10 |  | 1.02E-02 | 2.25E-03 | 2.30E-04 |
| 16 | rs6500437 | C | T | -3.55E-02 | 3.97E-03 | 4.60E-19 |  | -5.06E-03 | 1.99E-03 | 1.70E-02 |
| 10 | rs6584351 | G | A | 2.05E-02 | 3.68E-03 | 2.60E-08 |  | 4.04E-03 | 1.84E-03 | 2.50E-02 |
| 1 | rs6667957 | C | T | -2.60E-02 | 3.75E-03 | 4.30E-12 |  | -9.33E-04 | 1.86E-03 | 5.90E-01 |
| 2 | rs6736096 | C | T | 2.01E-02 | 3.68E-03 | 4.70E-08 |  | 2.16E-03 | 1.83E-03 | 4.40E-01 |
| 4 | rs6830848 | T | G | -3.48E-02 | 3.68E-03 | 3.60E-21 |  | 2.10E-04 | 1.85E-03 | 3.70E-01 |
| 6 | rs6912979 | C | T | -2.55E-02 | 4.09E-03 | 4.30E-10 |  | -7.90E-04 | 2.03E-03 | 9.60E-01 |
| 7 | rs6961014 | G | C | -2.90E-02 | 4.51E-03 | 1.20E-10 |  | -6.42E-03 | 2.23E-03 | 2.00E-03 |
| 10 | rs7087644 | G | A | -8.39E-02 | 9.31E-03 | 2.00E-19 |  | 3.53E-03 | 4.76E-03 | 3.90E-01 |
| 11 | rs7125555 | T | C | -2.76E-02 | 3.69E-03 | 6.50E-14 |  | -9.45E-04 | 1.84E-03 | 3.20E-01 |
| 1 | rs72708144 | C | T | 5.88E-02 | 9.21E-03 | 1.70E-10 |  | -1.87E-02 | 4.57E-03 | 4.00E-04 |
| 5 | rs72814771 | G | T | 4.94E-02 | 6.25E-03 | 2.90E-15 |  | -3.64E-03 | 3.12E-03 | 1.90E-01 |
| 2 | rs72827480 | C | T | 2.71E-02 | 3.76E-03 | 6.00E-13 |  | -4.14E-03 | 1.87E-03 | 1.70E-01 |
| 10 | rs728900 | A | T | -3.17E-02 | 3.76E-03 | 3.20E-17 |  | -7.56E-03 | 1.88E-03 | 1.60E-03 |
| 19 | rs73037453 | T | C | -2.64E-02 | 4.69E-03 | 1.70E-08 |  | -1.37E-02 | 2.36E-03 | 4.00E-07 |
| 20 | rs746748 | T | C | 4.74E-02 | 7.29E-03 | 7.50E-11 |  | 4.07E-03 | 3.64E-03 | 5.70E-01 |
| 10 | rs74701710 | A | G | -4.83E-02 | 8.04E-03 | 1.80E-09 |  | -8.97E-03 | 4.05E-03 | 2.90E-02 |
| 7 | rs74742883 | G | T | -3.11E-02 | 4.45E-03 | 3.00E-12 |  | -9.87E-03 | 2.21E-03 | 3.00E-06 |
| 12 | rs75770066 | G | A | 2.31E-01 | 1.06E-02 | 8.09E-105 |  | 2.60E-02 | 5.18E-03 | 4.90E-06 |
| 2 | rs7589040 | T | C | -3.15E-02 | 4.50E-03 | 2.40E-12 |  | -7.09E-04 | 2.28E-03 | 7.10E-01 |
| 4 | rs7661090 | T | C | -3.53E-02 | 5.96E-03 | 3.20E-09 |  | -4.70E-03 | 3.00E-03 | 2.10E-01 |
| 2 | rs76928871 | G | A | 4.30E-02 | 4.69E-03 | 4.30E-20 |  | 7.95E-03 | 2.32E-03 | 7.30E-04 |
| 12 | rs77100210 | C | A | 1.13E-01 | 8.44E-03 | 5.60E-41 |  | 8.98E-03 | 4.17E-03 | 3.50E-02 |
| 7 | rs7778113 | T | G | 2.80E-02 | 3.92E-03 | 9.40E-13 |  | 6.41E-04 | 1.94E-03 | 8.50E-01 |
| 1 | rs7779 | C | G | 3.99E-02 | 7.07E-03 | 1.70E-08 |  | 3.76E-04 | 3.52E-03 | 6.10E-01 |
| 8 | rs77952879 | C | G | -4.21E-02 | 6.95E-03 | 1.30E-09 |  | -9.38E-03 | 3.52E-03 | 4.50E-02 |
| 6 | rs78080415 | C | T | 2.98E-02 | 5.25E-03 | 1.30E-08 |  | -8.14E-03 | 2.61E-03 | 2.10E-03 |
| 8 | rs7827991 | A | C | 4.17E-02 | 6.88E-03 | 1.30E-09 |  | 1.56E-02 | 3.45E-03 | 2.80E-05 |
| 2 | rs78385274 | C | G | -5.94E-02 | 6.00E-03 | 4.60E-23 |  | -7.72E-03 | 2.99E-03 | 1.30E-02 |
| 2 | rs78385274 | C | G | -5.94E-02 | 6.00E-03 | 4.60E-23 |  | -4.10E-03 | 3.00E-03 | 1.20E-01 |
| 8 | rs7845046 | A | T | -4.92E-02 | 8.38E-03 | 4.40E-09 |  | 1.15E-02 | 4.25E-03 | 1.40E-03 |
| 16 | rs8045589 | T | A | -2.26E-02 | 3.70E-03 | 9.70E-10 |  | -4.33E-03 | 1.85E-03 | 4.20E-02 |
| 2 | rs809673 | G | A | -4.17E-02 | 3.77E-03 | 1.70E-28 |  | 3.60E-03 | 1.87E-03 | 5.50E-02 |
| 15 | rs888368 | G | A | 2.22E-02 | 3.68E-03 | 1.70E-09 |  | -4.84E-04 | 1.86E-03 | 6.70E-01 |
| 4 | rs9307242 | C | T | -2.72E-02 | 3.82E-03 | 1.20E-12 |  | 3.65E-03 | 1.94E-03 | 9.80E-02 |
| 5 | rs9313736 | A | G | -3.14E-02 | 3.77E-03 | 8.00E-17 |  | -1.41E-03 | 1.90E-03 | 1.30E-01 |
| 6 | rs9358956 | C | G | -6.59E-02 | 4.94E-03 | 1.60E-40 |  | -4.40E-03 | 2.44E-03 | 2.20E-01 |
| 1 | rs9438982 | A | C | -4.71E-02 | 3.95E-03 | 7.40E-33 |  | 1.18E-03 | 1.96E-03 | 4.80E-01 |
| 22 | rs9607474 | T | C | -3.24E-02 | 5.73E-03 | 1.50E-08 |  | -4.97E-03 | 2.89E-03 | 1.40E-01 |
| 22 | rs9613667 | C | A | -2.32E-02 | 3.88E-03 | 2.00E-09 |  | 3.33E-03 | 1.97E-03 | 1.10E-01 |
| 15 | rs9788714 | A | G | -4.29E-02 | 3.81E-03 | 1.80E-29 |  | 1.64E-03 | 1.92E-03 | 8.50E-01 |
| 3 | rs9818740 | A | G | -2.78E-02 | 4.14E-03 | 1.70E-11 |  | 5.03E-04 | 2.07E-03 | 9.30E-01 |
| 17 | rs9915489 | T | A | 4.36E-02 | 3.91E-03 | 7.20E-29 |  | 8.90E-03 | 1.96E-03 | 6.90E-04 |

Supplementary 15

| CHR | SNP | EA | OA | Age at menopause | | |  | Ultradistal forearm bone mineral density | | |
| --- | --- | --- | --- | --- | --- | --- | --- | --- | --- | --- |
|  |  |  |  | β | SE | P |  | β | SE | P |
| 11 | rs1020622 | G | C | 2.19E-02 | 3.73E-03 | 4.70E-09 |  | 1.98E-02 | 1.08E-02 | 6.74E-02 |
| 5 | rs10476835 | A | G | 2.11E-02 | 3.69E-03 | 1.00E-08 |  | 7.41E-03 | 1.06E-02 | 4.86E-01 |
| 9 | rs10813912 | G | A | 2.49E-02 | 3.80E-03 | 6.00E-11 |  | -1.38E-02 | 1.10E-02 | 2.13E-01 |
| 20 | rs10854167 | C | G | -3.95E-02 | 4.51E-03 | 1.90E-18 |  | 4.16E-03 | 1.28E-02 | 7.46E-01 |
| 3 | rs10937153 | A | G | 3.61E-02 | 4.33E-03 | 7.40E-17 |  | 4.23E-03 | 1.22E-02 | 7.28E-01 |
| 10 | rs10998203 | G | C | -3.61E-02 | 4.58E-03 | 3.30E-15 |  | 5.66E-03 | 1.34E-02 | 6.73E-01 |
| 11 | rs11031005 | C | T | 5.46E-02 | 5.27E-03 | 3.90E-25 |  | -1.26E-02 | 1.50E-02 | 3.98E-01 |
| 15 | rs112217463 | A | G | -3.73E-02 | 3.82E-03 | 1.50E-22 |  | -2.07E-02 | 1.14E-02 | 7.06E-02 |
| 13 | rs11571818 | C | T | -1.13E-01 | 1.91E-02 | 3.30E-09 |  | 6.72E-02 | 7.74E-02 | 3.85E-01 |
| 17 | rs11650324 | G | A | 3.91E-02 | 4.43E-03 | 1.10E-18 |  | 1.52E-02 | 1.28E-02 | 2.36E-01 |
| 1 | rs12046563 | G | A | -2.52E-02 | 4.32E-03 | 5.50E-09 |  | -3.57E-02 | 1.29E-02 | 5.53E-03 |
| 1 | rs12132692 | T | C | 4.03E-02 | 6.27E-03 | 1.30E-10 |  | 3.78E-03 | 1.60E-02 | 8.13E-01 |
| 4 | rs12503643 | T | G | 4.67E-02 | 3.77E-03 | 2.60E-35 |  | -3.46E-03 | 1.09E-02 | 7.51E-01 |
| 19 | rs12609254 | T | C | -3.39E-02 | 5.95E-03 | 1.20E-08 |  | 2.43E-03 | 1.67E-02 | 8.84E-01 |
| 4 | rs156520 | A | C | 2.25E-02 | 4.12E-03 | 4.90E-08 |  | 3.85E-02 | 1.23E-02 | 1.80E-03 |
| 4 | rs1565909 | T | C | 6.23E-02 | 3.69E-03 | 3.50E-64 |  | 2.21E-03 | 1.07E-02 | 8.36E-01 |
| 20 | rs16991615 | A | G | 2.43E-01 | 7.66E-03 | 1.00E-200 |  | 1.97E-02 | 1.89E-02 | 2.99E-01 |
| 14 | rs1760940 | C | A | -3.81E-02 | 4.26E-03 | 4.40E-19 |  | 1.08E-02 | 1.25E-02 | 3.88E-01 |
| 3 | rs17646517 | G | C | -7.16E-02 | 1.23E-02 | 5.10E-09 |  | 3.75E-02 | 3.63E-02 | 3.02E-01 |
| 16 | rs17680522 | G | A | 2.32E-02 | 4.06E-03 | 1.20E-08 |  | 1.50E-03 | 1.18E-02 | 8.99E-01 |
| 2 | rs17820747 | C | A | 2.60E-02 | 4.34E-03 | 2.10E-09 |  | -3.00E-03 | 1.35E-02 | 8.24E-01 |
| 14 | rs17856037 | T | C | -7.40E-02 | 1.17E-02 | 2.90E-10 |  | 3.28E-02 | 3.46E-02 | 3.43E-01 |
| 12 | rs1790123 | T | C | 3.47E-02 | 4.60E-03 | 4.70E-14 |  | -7.10E-03 | 1.39E-02 | 6.10E-01 |
| 12 | rs184540366 | T | G | -8.48E-02 | 1.29E-02 | 4.60E-11 |  | 4.36E-02 | 3.32E-02 | 1.89E-01 |
| 1 | rs200448 | C | T | -2.15E-02 | 3.73E-03 | 8.10E-09 |  | -9.36E-03 | 1.07E-02 | 3.82E-01 |
| 15 | rs2241522 | G | A | -2.16E-02 | 3.90E-03 | 2.90E-08 |  | -1.11E-02 | 1.13E-02 | 3.26E-01 |
| 12 | rs2277339 | G | T | -7.81E-02 | 6.00E-03 | 9.70E-39 |  | 3.51E-03 | 1.76E-02 | 8.42E-01 |
| 16 | rs251723 | C | G | 5.17E-02 | 3.88E-03 | 2.00E-40 |  | -5.11E-03 | 1.15E-02 | 6.58E-01 |
| 7 | rs2519673 | A | G | -2.68E-02 | 3.82E-03 | 2.40E-12 |  | -6.63E-03 | 1.08E-02 | 5.40E-01 |
| 3 | rs2624847 | T | G | 2.68E-02 | 4.22E-03 | 2.10E-10 |  | -2.38E-02 | 1.24E-02 | 5.56E-02 |
| 5 | rs2688194 | C | T | 4.41E-02 | 6.66E-03 | 3.60E-11 |  | 4.20E-03 | 2.10E-02 | 8.42E-01 |
| 12 | rs28416520 | A | G | -3.56E-02 | 3.73E-03 | 1.20E-21 |  | -7.95E-03 | 1.08E-02 | 4.61E-01 |
| 8 | rs28797500 | C | T | -8.46E-02 | 4.43E-03 | 3.80E-81 |  | -1.05E-02 | 1.29E-02 | 4.17E-01 |
| 17 | rs2941506 | G | A | 3.74E-02 | 3.97E-03 | 5.20E-21 |  | 6.61E-03 | 1.15E-02 | 5.66E-01 |
| 19 | rs299168 | A | G | 5.06E-02 | 6.29E-03 | 8.00E-16 |  | 1.60E-02 | 1.85E-02 | 3.87E-01 |
| 3 | rs345985 | T | C | -2.39E-02 | 3.75E-03 | 1.90E-10 |  | 6.30E-03 | 1.09E-02 | 5.65E-01 |
| 4 | rs34811474 | A | G | -2.39E-02 | 4.37E-03 | 4.50E-08 |  | -4.23E-03 | 1.24E-02 | 7.34E-01 |
| 19 | rs349306 | A | G | 5.25E-02 | 5.61E-03 | 8.60E-21 |  | 2.25E-02 | 1.61E-02 | 1.63E-01 |
| 19 | rs34962991 | A | G | -9.84E-02 | 3.83E-03 | 2.90E-145 |  | 8.90E-03 | 1.11E-02 | 4.24E-01 |
| 17 | rs3803756 | T | A | -2.48E-02 | 3.84E-03 | 1.20E-10 |  | -4.43E-03 | 1.09E-02 | 6.84E-01 |
| 19 | rs394448 | C | G | -2.05E-02 | 3.70E-03 | 3.20E-08 |  | -6.21E-03 | 1.07E-02 | 5.62E-01 |
| 7 | rs4049337 | C | G | -2.57E-02 | 4.02E-03 | 1.50E-10 |  | 3.32E-03 | 1.23E-02 | 7.87E-01 |
| 5 | rs419128 | A | G | -3.48E-02 | 3.86E-03 | 2.20E-19 |  | 6.23E-03 | 1.14E-02 | 5.85E-01 |
| 1 | rs4408133 | C | G | 3.16E-02 | 3.91E-03 | 6.90E-16 |  | 2.13E-02 | 1.14E-02 | 6.06E-02 |
| 2 | rs4491723 | G | A | 3.11E-02 | 4.18E-03 | 1.10E-13 |  | -5.48E-03 | 1.20E-02 | 6.47E-01 |
| 1 | rs4495657 | C | T | 3.28E-02 | 3.76E-03 | 2.40E-18 |  | -1.20E-02 | 1.10E-02 | 2.73E-01 |
| 2 | rs4668354 | G | C | 3.48E-02 | 3.80E-03 | 5.60E-20 |  | -2.93E-03 | 1.12E-02 | 7.93E-01 |
| 3 | rs4679121 | T | C | -3.81E-02 | 6.05E-03 | 3.10E-10 |  | -1.64E-02 | 1.75E-02 | 3.48E-01 |
| 6 | rs4716056 | G | A | 2.14E-02 | 3.79E-03 | 1.60E-08 |  | -1.46E-02 | 1.08E-02 | 1.78E-01 |
| 16 | rs4782369 | C | G | 3.00E-02 | 3.93E-03 | 2.10E-14 |  | -1.59E-02 | 1.13E-02 | 1.59E-01 |
| 22 | rs4821798 | C | T | -4.35E-02 | 3.99E-03 | 1.20E-27 |  | -2.09E-02 | 1.16E-02 | 7.05E-02 |
| 13 | rs4886238 | A | G | 3.80E-02 | 3.95E-03 | 6.30E-22 |  | 1.26E-02 | 1.13E-02 | 2.64E-01 |
| 3 | rs507926 | C | T | 2.99E-02 | 4.94E-03 | 1.50E-09 |  | 7.53E-03 | 1.37E-02 | 5.82E-01 |
| 20 | rs536092 | T | C | 2.38E-02 | 3.81E-03 | 3.90E-10 |  | 6.22E-03 | 1.08E-02 | 5.64E-01 |
| 1 | rs55707872 | C | A | 3.54E-02 | 3.98E-03 | 5.70E-19 |  | 9.62E-03 | 1.15E-02 | 4.02E-01 |
| 15 | rs55848327 | A | G | 2.52E-02 | 4.37E-03 | 8.80E-09 |  | 1.45E-02 | 1.26E-02 | 2.49E-01 |
| 5 | rs58279426 | C | T | 6.76E-02 | 3.70E-03 | 1.50E-74 |  | 5.85E-03 | 1.07E-02 | 5.83E-01 |
| 20 | rs6011452 | A | C | -4.91E-02 | 4.45E-03 | 2.80E-28 |  | -3.00E-05 | 1.37E-02 | 9.98E-01 |
| 19 | rs60907808 | G | A | -4.44E-02 | 5.26E-03 | 3.20E-17 |  | -3.25E-02 | 1.47E-02 | 2.65E-02 |
| 20 | rs6139074 | C | A | -3.06E-02 | 4.56E-03 | 1.80E-11 |  | -2.59E-02 | 1.31E-02 | 4.85E-02 |
| 2 | rs62156695 | G | A | -6.41E-02 | 6.04E-03 | 2.70E-26 |  | 1.25E-02 | 1.79E-02 | 4.84E-01 |
| 7 | rs62445870 | T | C | 7.46E-02 | 1.24E-02 | 1.90E-09 |  | -1.90E-02 | 3.41E-02 | 5.78E-01 |
| 2 | rs6435156 | T | C | -2.63E-02 | 4.22E-03 | 4.50E-10 |  | -9.48E-03 | 1.21E-02 | 4.32E-01 |
| 8 | rs6470643 | C | A | -2.83E-02 | 4.47E-03 | 2.40E-10 |  | 2.70E-02 | 1.31E-02 | 3.93E-02 |
| 16 | rs6500437 | C | T | -3.55E-02 | 3.97E-03 | 4.60E-19 |  | -4.85E-03 | 1.14E-02 | 6.70E-01 |
| 10 | rs6584351 | G | A | 2.05E-02 | 3.68E-03 | 2.60E-08 |  | -4.02E-03 | 1.07E-02 | 7.07E-01 |
| 1 | rs6667957 | C | T | -2.60E-02 | 3.75E-03 | 4.30E-12 |  | -2.12E-03 | 1.10E-02 | 8.47E-01 |
| 2 | rs6736096 | C | T | 2.01E-02 | 3.68E-03 | 4.70E-08 |  | 9.65E-03 | 1.07E-02 | 3.67E-01 |
| 4 | rs6830848 | T | G | -3.48E-02 | 3.68E-03 | 3.60E-21 |  | -7.02E-03 | 1.06E-02 | 5.10E-01 |
| 6 | rs6912979 | C | T | -2.55E-02 | 4.09E-03 | 4.30E-10 |  | -1.35E-02 | 1.18E-02 | 2.51E-01 |
| 7 | rs6961014 | G | C | -2.90E-02 | 4.51E-03 | 1.20E-10 |  | -3.25E-02 | 1.31E-02 | 1.31E-02 |
| 10 | rs7087644 | G | A | -8.39E-02 | 9.31E-03 | 2.00E-19 |  | 2.24E-03 | 2.98E-02 | 9.40E-01 |
| 11 | rs7125555 | T | C | -2.76E-02 | 3.69E-03 | 6.50E-14 |  | -3.17E-03 | 1.08E-02 | 7.68E-01 |
| 1 | rs72708144 | C | T | 5.88E-02 | 9.21E-03 | 1.70E-10 |  | -3.28E-02 | 3.23E-02 | 3.10E-01 |
| 2 | rs72827480 | C | T | 2.71E-02 | 3.76E-03 | 6.00E-13 |  | 3.66E-03 | 1.08E-02 | 7.34E-01 |
| 10 | rs728900 | A | T | -3.17E-02 | 3.76E-03 | 3.20E-17 |  | -1.25E-03 | 1.09E-02 | 9.08E-01 |
| 19 | rs73037453 | T | C | -2.64E-02 | 4.69E-03 | 1.70E-08 |  | -3.80E-03 | 1.34E-02 | 7.76E-01 |
| 20 | rs746748 | T | C | 4.74E-02 | 7.29E-03 | 7.50E-11 |  | 1.96E-02 | 2.16E-02 | 3.64E-01 |
| 10 | rs74701710 | A | G | -4.83E-02 | 8.04E-03 | 1.80E-09 |  | 9.23E-03 | 2.71E-02 | 7.33E-01 |
| 7 | rs74742883 | G | T | -3.11E-02 | 4.45E-03 | 3.00E-12 |  | -2.03E-02 | 1.30E-02 | 1.19E-01 |
| 12 | rs75770066 | G | A | 2.31E-01 | 1.06E-02 | 8.09E-105 |  | 3.36E-02 | 3.23E-02 | 2.99E-01 |
| 2 | rs7589040 | T | C | -3.15E-02 | 4.50E-03 | 2.40E-12 |  | 6.48E-03 | 1.47E-02 | 6.60E-01 |
| 4 | rs7661090 | T | C | -3.53E-02 | 5.96E-03 | 3.20E-09 |  | 2.07E-02 | 1.84E-02 | 2.61E-01 |
| 2 | rs76928871 | G | A | 4.30E-02 | 4.69E-03 | 4.30E-20 |  | 1.59E-02 | 1.47E-02 | 2.82E-01 |
| 12 | rs77100210 | C | A | 1.13E-01 | 8.44E-03 | 5.60E-41 |  | 2.79E-02 | 2.28E-02 | 2.20E-01 |
| 7 | rs7778113 | T | G | 2.80E-02 | 3.92E-03 | 9.40E-13 |  | -5.34E-03 | 1.10E-02 | 6.29E-01 |
| 1 | rs7779 | C | G | 3.99E-02 | 7.07E-03 | 1.70E-08 |  | 5.90E-03 | 2.77E-02 | 8.31E-01 |
| 8 | rs77952879 | C | G | -4.21E-02 | 6.95E-03 | 1.30E-09 |  | -2.04E-02 | 1.94E-02 | 2.93E-01 |
| 6 | rs78080415 | C | T | 2.98E-02 | 5.25E-03 | 1.30E-08 |  | -3.60E-04 | 1.57E-02 | 9.82E-01 |
| 8 | rs7827991 | A | C | 4.17E-02 | 6.88E-03 | 1.30E-09 |  | 3.00E-02 | 2.04E-02 | 1.41E-01 |
| 2 | rs78385274 | C | G | -5.94E-02 | 6.00E-03 | 4.60E-23 |  | 2.02E-02 | 1.82E-02 | 2.68E-01 |
| 2 | rs78385274 | C | G | -5.94E-02 | 6.00E-03 | 4.60E-23 |  | -2.95E-03 | 1.81E-02 | 8.70E-01 |
| 8 | rs7845046 | A | T | -4.92E-02 | 8.38E-03 | 4.40E-09 |  | 2.04E-02 | 2.23E-02 | 3.61E-01 |
| 16 | rs8045589 | T | A | -2.26E-02 | 3.70E-03 | 9.70E-10 |  | 6.31E-04 | 1.07E-02 | 9.53E-01 |
| 2 | rs809673 | G | A | -4.17E-02 | 3.77E-03 | 1.70E-28 |  | -1.73E-02 | 1.07E-02 | 1.06E-01 |
| 15 | rs888368 | G | A | 2.22E-02 | 3.68E-03 | 1.70E-09 |  | 1.52E-02 | 1.07E-02 | 1.53E-01 |
| 4 | rs9307242 | C | T | -2.72E-02 | 3.82E-03 | 1.20E-12 |  | 2.06E-02 | 1.14E-02 | 7.07E-02 |
| 5 | rs9313736 | A | G | -3.14E-02 | 3.77E-03 | 8.00E-17 |  | -3.32E-02 | 1.10E-02 | 2.49E-03 |
| 6 | rs9358956 | C | G | -6.59E-02 | 4.94E-03 | 1.60E-40 |  | -2.98E-02 | 1.43E-02 | 3.67E-02 |
| 1 | rs9438982 | A | C | -4.71E-02 | 3.95E-03 | 7.40E-33 |  | -7.31E-03 | 1.12E-02 | 5.14E-01 |
| 22 | rs9607474 | T | C | -3.24E-02 | 5.73E-03 | 1.50E-08 |  | -1.45E-02 | 1.69E-02 | 3.94E-01 |
| 22 | rs9613667 | C | A | -2.32E-02 | 3.88E-03 | 2.00E-09 |  | -1.03E-02 | 1.12E-02 | 3.57E-01 |
| 15 | rs9788714 | A | G | -4.29E-02 | 3.81E-03 | 1.80E-29 |  | -3.15E-03 | 1.12E-02 | 7.78E-01 |
| 3 | rs9818740 | A | G | -2.78E-02 | 4.14E-03 | 1.70E-11 |  | 2.82E-03 | 1.20E-02 | 8.14E-01 |
| 17 | rs9915489 | T | A | 4.36E-02 | 3.91E-03 | 7.20E-29 |  | 1.24E-02 | 1.15E-02 | 2.81E-01 |

Supplementary 16

| CHR | SNP | EA | OA | Age at menopause | | |  | Lumbar spine bone mineral density | | |
| --- | --- | --- | --- | --- | --- | --- | --- | --- | --- | --- |
|  |  |  |  | β | SE | P |  | β | SE | P |
| 11 | rs1020622 | G | C | 2.19E-02 | 3.73E-03 | 4.70E-09 |  | 1.83E-02 | 8.77E-03 | 4.14E-02 |
| 5 | rs10476835 | A | G | 2.11E-02 | 3.69E-03 | 1.00E-08 |  | 1.57E-02 | 8.70E-03 | 7.88E-02 |
| 16 | rs10521305 | C | T | 5.44E-02 | 7.78E-03 | 2.70E-12 |  | 1.64E-02 | 1.96E-02 | 4.13E-01 |
| 9 | rs10813912 | G | A | 2.49E-02 | 3.80E-03 | 6.00E-11 |  | -1.46E-02 | 9.02E-03 | 1.14E-01 |
| 20 | rs10854167 | C | G | -3.95E-02 | 4.51E-03 | 1.90E-18 |  | 2.15E-03 | 1.07E-02 | 8.44E-01 |
| 3 | rs10937153 | A | G | 3.61E-02 | 4.33E-03 | 7.40E-17 |  | 1.96E-02 | 1.02E-02 | 6.16E-02 |
| 10 | rs10998203 | G | C | -3.61E-02 | 4.58E-03 | 3.30E-15 |  | -4.04E-03 | 1.09E-02 | 7.16E-01 |
| 11 | rs11031005 | C | T | 5.46E-02 | 5.27E-03 | 3.90E-25 |  | -2.98E-02 | 1.23E-02 | 1.83E-02 |
| 7 | rs112190116 | T | C | 1.43E-01 | 1.76E-02 | 5.10E-16 |  | -2.63E-02 | 5.63E-02 | 6.48E-01 |
| 15 | rs112217463 | A | G | -3.73E-02 | 3.82E-03 | 1.50E-22 |  | 1.28E-03 | 9.16E-03 | 8.92E-01 |
| 17 | rs11650324 | G | A | 3.91E-02 | 4.43E-03 | 1.10E-18 |  | -2.37E-03 | 1.05E-02 | 8.26E-01 |
| 1 | rs12046563 | G | A | -2.52E-02 | 4.32E-03 | 5.50E-09 |  | -2.24E-02 | 1.01E-02 | 3.12E-02 |
| 1 | rs12132692 | T | C | 4.03E-02 | 6.27E-03 | 1.30E-10 |  | -1.68E-02 | 1.46E-02 | 2.59E-01 |
| 16 | rs12444283 | G | C | -2.89E-02 | 3.96E-03 | 2.70E-13 |  | -1.60E-04 | 9.44E-03 | 9.87E-01 |
| 4 | rs12503643 | T | G | 4.67E-02 | 3.77E-03 | 2.60E-35 |  | 1.34E-02 | 8.88E-03 | 1.40E-01 |
| 19 | rs12609254 | T | C | -3.39E-02 | 5.95E-03 | 1.20E-08 |  | 6.49E-03 | 1.41E-02 | 6.54E-01 |
| 4 | rs156520 | A | C | 2.25E-02 | 4.12E-03 | 4.90E-08 |  | 1.20E-03 | 9.59E-03 | 9.03E-01 |
| 4 | rs1565909 | T | C | 6.23E-02 | 3.69E-03 | 3.50E-64 |  | -5.32E-03 | 8.79E-03 | 5.55E-01 |
| 6 | rs1655907 | C | T | -2.90E-02 | 5.09E-03 | 1.10E-08 |  | 8.07E-03 | 1.64E-02 | 6.30E-01 |
| 14 | rs1760940 | C | A | -3.81E-02 | 4.26E-03 | 4.40E-19 |  | 2.48E-03 | 1.03E-02 | 8.15E-01 |
| 17 | rs17650301 | C | A | -4.01E-02 | 4.04E-03 | 2.80E-23 |  | -2.30E-02 | 9.99E-03 | 2.47E-02 |
| 16 | rs17680522 | G | A | 2.32E-02 | 4.06E-03 | 1.20E-08 |  | 8.22E-03 | 9.61E-03 | 4.03E-01 |
| 2 | rs17820747 | C | A | 2.60E-02 | 4.34E-03 | 2.10E-09 |  | -4.06E-03 | 1.03E-02 | 7.00E-01 |
| 14 | rs17856037 | T | C | -7.40E-02 | 1.17E-02 | 2.90E-10 |  | -2.71E-02 | 2.81E-02 | 3.46E-01 |
| 12 | rs1790123 | T | C | 3.47E-02 | 4.60E-03 | 4.70E-14 |  | 7.34E-03 | 1.10E-02 | 5.15E-01 |
| 12 | rs184540366 | T | G | -8.48E-02 | 1.29E-02 | 4.60E-11 |  | 5.83E-02 | 3.50E-02 | 1.04E-01 |
| 1 | rs200448 | C | T | -2.15E-02 | 3.73E-03 | 8.10E-09 |  | -6.03E-03 | 8.83E-03 | 5.05E-01 |
| 16 | rs251723 | C | G | 5.17E-02 | 3.88E-03 | 2.00E-40 |  | -4.40E-03 | 9.18E-03 | 6.39E-01 |
| 7 | rs2519673 | A | G | -2.68E-02 | 3.82E-03 | 2.40E-12 |  | 1.96E-03 | 9.01E-03 | 8.31E-01 |
| 6 | rs2524119 | C | T | 2.38E-02 | 3.69E-03 | 1.20E-10 |  | -6.30E-03 | 1.23E-02 | 6.17E-01 |
| 5 | rs2688194 | C | T | 4.41E-02 | 6.66E-03 | 3.60E-11 |  | 3.66E-03 | 1.55E-02 | 8.17E-01 |
| 12 | rs28416520 | A | G | -3.56E-02 | 3.73E-03 | 1.20E-21 |  | 5.95E-03 | 8.80E-03 | 5.09E-01 |
| 6 | rs2844466 | C | T | -4.72E-02 | 3.84E-03 | 8.30E-35 |  | 2.07E-03 | 9.84E-03 | 8.37E-01 |
| 8 | rs28797500 | C | T | -8.46E-02 | 4.43E-03 | 3.80E-81 |  | 2.47E-03 | 1.13E-02 | 8.32E-01 |
| 17 | rs2941506 | G | A | 3.74E-02 | 3.97E-03 | 5.20E-21 |  | 7.60E-04 | 1.11E-02 | 9.46E-01 |
| 19 | rs299168 | A | G | 5.06E-02 | 6.29E-03 | 8.00E-16 |  | 8.27E-03 | 1.50E-02 | 5.91E-01 |
| 3 | rs345985 | T | C | -2.39E-02 | 3.75E-03 | 1.90E-10 |  | -2.39E-02 | 8.84E-03 | 8.44E-03 |
| 19 | rs349306 | A | G | 5.25E-02 | 5.61E-03 | 8.60E-21 |  | -3.99E-03 | 1.37E-02 | 7.76E-01 |
| 19 | rs34962991 | A | G | -9.84E-02 | 3.83E-03 | 2.90E-145 |  | -1.79E-02 | 9.13E-03 | 5.55E-02 |
| 17 | rs3803756 | T | A | -2.48E-02 | 3.84E-03 | 1.20E-10 |  | 3.39E-03 | 9.16E-03 | 7.18E-01 |
| 19 | rs394448 | C | G | -2.05E-02 | 3.70E-03 | 3.20E-08 |  | -1.35E-02 | 8.74E-03 | 1.32E-01 |
| 7 | rs4049337 | C | G | -2.57E-02 | 4.02E-03 | 1.50E-10 |  | -2.15E-02 | 9.55E-03 | 2.82E-02 |
| 5 | rs419128 | A | G | -3.48E-02 | 3.86E-03 | 2.20E-19 |  | -2.76E-02 | 9.13E-03 | 3.15E-03 |
| 1 | rs4408133 | C | G | 3.16E-02 | 3.91E-03 | 6.90E-16 |  | -6.82E-03 | 9.46E-03 | 4.81E-01 |
| 2 | rs4491723 | G | A | 3.11E-02 | 4.18E-03 | 1.10E-13 |  | -6.13E-03 | 9.85E-03 | 5.44E-01 |
| 1 | rs4495657 | C | T | 3.28E-02 | 3.76E-03 | 2.40E-18 |  | 1.76E-02 | 8.85E-03 | 5.28E-02 |
| 2 | rs4668354 | G | C | 3.48E-02 | 3.80E-03 | 5.60E-20 |  | 2.25E-03 | 9.10E-03 | 8.09E-01 |
| 6 | rs4716056 | G | A | 2.14E-02 | 3.79E-03 | 1.60E-08 |  | 3.91E-03 | 8.94E-03 | 6.69E-01 |
| 16 | rs4782369 | C | G | 3.00E-02 | 3.93E-03 | 2.10E-14 |  | -1.71E-02 | 9.95E-03 | 9.29E-02 |
| 22 | rs4821798 | C | T | -4.35E-02 | 3.99E-03 | 1.20E-27 |  | -3.05E-02 | 1.14E-02 | 9.07E-03 |
| 13 | rs4886238 | A | G | 3.80E-02 | 3.95E-03 | 6.30E-22 |  | 1.72E-02 | 9.36E-03 | 7.26E-02 |
| 3 | rs507926 | C | T | 2.99E-02 | 4.94E-03 | 1.50E-09 |  | -6.71E-03 | 1.19E-02 | 5.81E-01 |
| 20 | rs536092 | T | C | 2.38E-02 | 3.81E-03 | 3.90E-10 |  | 7.52E-03 | 9.08E-03 | 4.19E-01 |
| 1 | rs55707872 | C | A | 3.54E-02 | 3.98E-03 | 5.70E-19 |  | 1.78E-02 | 9.62E-03 | 7.08E-02 |
| 15 | rs55848327 | A | G | 2.52E-02 | 4.37E-03 | 8.80E-09 |  | 1.05E-03 | 1.16E-02 | 9.29E-01 |
| 5 | rs58279426 | C | T | 6.76E-02 | 3.70E-03 | 1.50E-74 |  | -4.98E-03 | 8.68E-03 | 5.75E-01 |
| 19 | rs60907808 | G | A | -4.44E-02 | 5.26E-03 | 3.20E-17 |  | -2.85E-02 | 1.27E-02 | 2.87E-02 |
| 20 | rs6139074 | C | A | -3.06E-02 | 4.56E-03 | 1.80E-11 |  | -8.85E-03 | 1.14E-02 | 4.48E-01 |
| 10 | rs61870304 | G | A | -3.68E-02 | 6.11E-03 | 1.70E-09 |  | 2.30E-02 | 1.51E-02 | 1.36E-01 |
| 2 | rs62156695 | G | A | -6.41E-02 | 6.04E-03 | 2.70E-26 |  | -2.31E-02 | 1.44E-02 | 1.19E-01 |
| 2 | rs6435156 | T | C | -2.63E-02 | 4.22E-03 | 4.50E-10 |  | -1.98E-02 | 1.01E-02 | 5.56E-02 |
| 8 | rs6470643 | C | A | -2.83E-02 | 4.47E-03 | 2.40E-10 |  | -1.87E-02 | 1.06E-02 | 8.45E-02 |
| 16 | rs6500437 | C | T | -3.55E-02 | 3.97E-03 | 4.60E-19 |  | -7.42E-03 | 9.60E-03 | 4.50E-01 |
| 10 | rs6584351 | G | A | 2.05E-02 | 3.68E-03 | 2.60E-08 |  | 6.68E-03 | 8.73E-03 | 4.55E-01 |
| 1 | rs6667957 | C | T | -2.60E-02 | 3.75E-03 | 4.30E-12 |  | 4.48E-03 | 8.80E-03 | 6.19E-01 |
| 2 | rs6736096 | C | T | 2.01E-02 | 3.68E-03 | 4.70E-08 |  | 1.11E-02 | 8.69E-03 | 2.11E-01 |
| 4 | rs6830848 | T | G | -3.48E-02 | 3.68E-03 | 3.60E-21 |  | -2.09E-02 | 8.98E-03 | 2.31E-02 |
| 6 | rs6912979 | C | T | -2.55E-02 | 4.09E-03 | 4.30E-10 |  | -1.96E-03 | 9.83E-03 | 8.46E-01 |
| 7 | rs6961014 | G | C | -2.90E-02 | 4.51E-03 | 1.20E-10 |  | -6.43E-03 | 1.08E-02 | 5.62E-01 |
| 10 | rs7087644 | G | A | -8.39E-02 | 9.31E-03 | 2.00E-19 |  | -1.70E-02 | 2.15E-02 | 4.40E-01 |
| 11 | rs7125555 | T | C | -2.76E-02 | 3.69E-03 | 6.50E-14 |  | -1.54E-02 | 8.74E-03 | 8.53E-02 |
| 1 | rs72708144 | C | T | 5.88E-02 | 9.21E-03 | 1.70E-10 |  | 4.51E-02 | 2.31E-02 | 5.66E-02 |
| 5 | rs72814771 | G | T | 4.94E-02 | 6.25E-03 | 2.90E-15 |  | 4.47E-03 | 1.42E-02 | 7.59E-01 |
| 2 | rs72827480 | C | T | 2.71E-02 | 3.76E-03 | 6.00E-13 |  | -1.70E-02 | 9.13E-03 | 6.86E-02 |
| 10 | rs728900 | A | T | -3.17E-02 | 3.76E-03 | 3.20E-17 |  | -1.93E-03 | 8.85E-03 | 8.32E-01 |
| 19 | rs73037453 | T | C | -2.64E-02 | 4.69E-03 | 1.70E-08 |  | -3.35E-02 | 1.12E-02 | 3.61E-03 |
| 20 | rs746748 | T | C | 4.74E-02 | 7.29E-03 | 7.50E-11 |  | -1.77E-02 | 1.78E-02 | 3.32E-01 |
| 10 | rs74701710 | A | G | -4.83E-02 | 8.04E-03 | 1.80E-09 |  | 8.93E-04 | 1.88E-02 | 9.63E-01 |
| 7 | rs74742883 | G | T | -3.11E-02 | 4.45E-03 | 3.00E-12 |  | -1.30E-03 | 1.05E-02 | 9.03E-01 |
| 2 | rs7589040 | T | C | -3.15E-02 | 4.50E-03 | 2.40E-12 |  | -1.23E-02 | 1.02E-02 | 2.37E-01 |
| 4 | rs7661090 | T | C | -3.53E-02 | 5.96E-03 | 3.20E-09 |  | -1.51E-03 | 1.38E-02 | 9.15E-01 |
| 2 | rs76928871 | G | A | 4.30E-02 | 4.69E-03 | 4.30E-20 |  | 2.63E-02 | 1.12E-02 | 2.10E-02 |
| 7 | rs7778113 | T | G | 2.80E-02 | 3.92E-03 | 9.40E-13 |  | 7.85E-03 | 1.02E-02 | 4.54E-01 |
| 8 | rs77952879 | C | G | -4.21E-02 | 6.95E-03 | 1.30E-09 |  | 2.84E-02 | 1.83E-02 | 1.30E-01 |
| 6 | rs78080415 | C | T | 2.98E-02 | 5.25E-03 | 1.30E-08 |  | 7.31E-03 | 1.24E-02 | 5.67E-01 |
| 8 | rs7827991 | A | C | 4.17E-02 | 6.88E-03 | 1.30E-09 |  | -2.25E-02 | 1.63E-02 | 1.76E-01 |
| 2 | rs78385274 | C | G | -5.94E-02 | 6.00E-03 | 4.60E-23 |  | 1.69E-02 | 1.34E-02 | 2.18E-01 |
| 8 | rs7845046 | A | T | -4.92E-02 | 8.38E-03 | 4.40E-09 |  | 1.99E-02 | 2.02E-02 | 3.37E-01 |
| 16 | rs8045589 | T | A | -2.26E-02 | 3.70E-03 | 9.70E-10 |  | -5.22E-03 | 8.73E-03 | 5.59E-01 |
| 2 | rs809673 | G | A | -4.17E-02 | 3.77E-03 | 1.70E-28 |  | 2.23E-02 | 9.15E-03 | 1.73E-02 |
| 15 | rs888368 | G | A | 2.22E-02 | 3.68E-03 | 1.70E-09 |  | 1.35E-02 | 8.74E-03 | 1.31E-01 |
| 4 | rs9307242 | C | T | -2.72E-02 | 3.82E-03 | 1.20E-12 |  | 8.93E-03 | 9.08E-03 | 3.37E-01 |
| 5 | rs9313736 | A | G | -3.14E-02 | 3.77E-03 | 8.00E-17 |  | -7.04E-03 | 8.95E-03 | 4.42E-01 |
| 1 | rs9438982 | A | C | -4.71E-02 | 3.95E-03 | 7.40E-33 |  | -3.18E-03 | 9.19E-03 | 7.35E-01 |
| 22 | rs9607474 | T | C | -3.24E-02 | 5.73E-03 | 1.50E-08 |  | 1.07E-02 | 1.58E-02 | 5.11E-01 |
| 22 | rs9613667 | C | A | -2.32E-02 | 3.88E-03 | 2.00E-09 |  | 7.69E-03 | 1.12E-02 | 5.02E-01 |
| 15 | rs9788714 | A | G | -4.29E-02 | 3.81E-03 | 1.80E-29 |  | -9.86E-04 | 9.10E-03 | 9.16E-01 |
| 3 | rs9818740 | A | G | -2.78E-02 | 4.14E-03 | 1.70E-11 |  | 4.48E-04 | 9.98E-03 | 9.65E-01 |
| 17 | rs9915489 | T | A | 4.36E-02 | 3.91E-03 | 7.20E-29 |  | 4.58E-03 | 9.37E-03 | 6.33E-01 |

Supplementary 17

| CHR | SNP | EA | OA | Age at menopause | | |  | Femoral neck bone mineral density | | |
| --- | --- | --- | --- | --- | --- | --- | --- | --- | --- | --- |
|  |  |  |  | β | SE | P |  | β | SE | P |
| 11 | rs1020622 | G | C | 2.19E-02 | 3.73E-03 | 4.70E-09 |  | 1.13E-02 | 7.53E-03 | 1.41E-01 |
| 5 | rs10476835 | A | G | 2.11E-02 | 3.69E-03 | 1.00E-08 |  | 6.29E-03 | 7.48E-03 | 4.10E-01 |
| 16 | rs10521305 | C | T | 5.44E-02 | 7.78E-03 | 2.70E-12 |  | 2.83E-02 | 1.69E-02 | 1.01E-01 |
| 9 | rs10813912 | G | A | 2.49E-02 | 3.80E-03 | 6.00E-11 |  | -4.38E-03 | 7.76E-03 | 5.81E-01 |
| 20 | rs10854167 | C | G | -3.95E-02 | 4.51E-03 | 1.90E-18 |  | -1.49E-02 | 9.19E-03 | 1.12E-01 |
| 3 | rs10937153 | A | G | 3.61E-02 | 4.33E-03 | 7.40E-17 |  | 1.52E-02 | 8.75E-03 | 8.95E-02 |
| 10 | rs10998203 | G | C | -3.61E-02 | 4.58E-03 | 3.30E-15 |  | -1.04E-02 | 9.33E-03 | 2.76E-01 |
| 11 | rs11031005 | C | T | 5.46E-02 | 5.27E-03 | 3.90E-25 |  | -2.36E-02 | 1.06E-02 | 3.01E-02 |
| 7 | rs112190116 | T | C | 1.43E-01 | 1.76E-02 | 5.10E-16 |  | 2.02E-02 | 4.74E-02 | 6.77E-01 |
| 15 | rs112217463 | A | G | -3.73E-02 | 3.82E-03 | 1.50E-22 |  | -7.74E-03 | 7.86E-03 | 3.35E-01 |
| 17 | rs11650324 | G | A | 3.91E-02 | 4.43E-03 | 1.10E-18 |  | -4.31E-03 | 9.02E-03 | 6.40E-01 |
| 1 | rs12046563 | G | A | -2.52E-02 | 4.32E-03 | 5.50E-09 |  | -2.18E-03 | 8.71E-03 | 8.06E-01 |
| 1 | rs12132692 | T | C | 4.03E-02 | 6.27E-03 | 1.30E-10 |  | -2.26E-02 | 1.25E-02 | 7.74E-02 |
| 16 | rs12444283 | G | C | -2.89E-02 | 3.96E-03 | 2.70E-13 |  | -8.79E-03 | 8.13E-03 | 2.90E-01 |
| 4 | rs12503643 | T | G | 4.67E-02 | 3.77E-03 | 2.60E-35 |  | 2.28E-03 | 7.64E-03 | 7.71E-01 |
| 19 | rs12609254 | T | C | -3.39E-02 | 5.95E-03 | 1.20E-08 |  | 9.69E-03 | 1.22E-02 | 4.37E-01 |
| 4 | rs156520 | A | C | 2.25E-02 | 4.12E-03 | 4.90E-08 |  | -5.22E-03 | 8.27E-03 | 5.37E-01 |
| 4 | rs1565909 | T | C | 6.23E-02 | 3.69E-03 | 3.50E-64 |  | 5.22E-03 | 7.54E-03 | 4.98E-01 |
| 6 | rs1655907 | C | T | -2.90E-02 | 5.09E-03 | 1.10E-08 |  | -3.97E-03 | 1.29E-02 | 7.64E-01 |
| 14 | rs1760940 | C | A | -3.81E-02 | 4.26E-03 | 4.40E-19 |  | 1.42E-02 | 8.93E-03 | 1.20E-01 |
| 17 | rs17650301 | C | A | -4.01E-02 | 4.04E-03 | 2.80E-23 |  | -1.04E-02 | 8.67E-03 | 2.40E-01 |
| 16 | rs17680522 | G | A | 2.32E-02 | 4.06E-03 | 1.20E-08 |  | 2.47E-03 | 8.24E-03 | 7.69E-01 |
| 2 | rs17820747 | C | A | 2.60E-02 | 4.34E-03 | 2.10E-09 |  | 2.30E-03 | 8.87E-03 | 8.00E-01 |
| 14 | rs17856037 | T | C | -7.40E-02 | 1.17E-02 | 2.90E-10 |  | -4.85E-02 | 2.45E-02 | 5.28E-02 |
| 12 | rs1790123 | T | C | 3.47E-02 | 4.60E-03 | 4.70E-14 |  | 2.44E-03 | 9.47E-03 | 8.01E-01 |
| 12 | rs184540366 | T | G | -8.48E-02 | 1.29E-02 | 4.60E-11 |  | 1.55E-02 | 3.01E-02 | 6.13E-01 |
| 1 | rs200448 | C | T | -2.15E-02 | 3.73E-03 | 8.10E-09 |  | -1.39E-02 | 7.58E-03 | 7.23E-02 |
| 16 | rs251723 | C | G | 5.17E-02 | 3.88E-03 | 2.00E-40 |  | -6.01E-03 | 7.90E-03 | 4.57E-01 |
| 7 | rs2519673 | A | G | -2.68E-02 | 3.82E-03 | 2.40E-12 |  | 6.27E-03 | 7.71E-03 | 4.26E-01 |
| 6 | rs2524119 | C | T | 2.38E-02 | 3.69E-03 | 1.20E-10 |  | -9.28E-03 | 1.08E-02 | 3.99E-01 |
| 5 | rs2688194 | C | T | 4.41E-02 | 6.66E-03 | 3.60E-11 |  | 1.47E-04 | 1.34E-02 | 9.91E-01 |
| 12 | rs28416520 | A | G | -3.56E-02 | 3.73E-03 | 1.20E-21 |  | 2.78E-03 | 7.58E-03 | 7.19E-01 |
| 6 | rs2844466 | C | T | -4.72E-02 | 3.84E-03 | 8.30E-35 |  | 9.48E-03 | 8.32E-03 | 2.65E-01 |
| 8 | rs28797500 | C | T | -8.46E-02 | 4.43E-03 | 3.80E-81 |  | 6.77E-03 | 9.56E-03 | 4.88E-01 |
| 17 | rs2941506 | G | A | 3.74E-02 | 3.97E-03 | 5.20E-21 |  | -7.72E-03 | 9.05E-03 | 4.04E-01 |
| 19 | rs299168 | A | G | 5.06E-02 | 6.29E-03 | 8.00E-16 |  | -8.15E-04 | 1.30E-02 | 9.51E-01 |
| 3 | rs345985 | T | C | -2.39E-02 | 3.75E-03 | 1.90E-10 |  | -1.45E-02 | 7.59E-03 | 6.12E-02 |
| 19 | rs349306 | A | G | 5.25E-02 | 5.61E-03 | 8.60E-21 |  | 2.36E-02 | 1.19E-02 | 5.22E-02 |
| 19 | rs34962991 | A | G | -9.84E-02 | 3.83E-03 | 2.90E-145 |  | 1.20E-03 | 7.84E-03 | 8.81E-01 |
| 17 | rs3803756 | T | A | -2.48E-02 | 3.84E-03 | 1.20E-10 |  | -2.31E-03 | 7.87E-03 | 7.74E-01 |
| 19 | rs394448 | C | G | -2.05E-02 | 3.70E-03 | 3.20E-08 |  | -1.21E-02 | 7.50E-03 | 1.13E-01 |
| 7 | rs4049337 | C | G | -2.57E-02 | 4.02E-03 | 1.50E-10 |  | -4.42E-03 | 8.13E-03 | 5.95E-01 |
| 5 | rs419128 | A | G | -3.48E-02 | 3.86E-03 | 2.20E-19 |  | -9.16E-03 | 7.86E-03 | 2.54E-01 |
| 1 | rs4408133 | C | G | 3.16E-02 | 3.91E-03 | 6.90E-16 |  | 8.62E-04 | 8.11E-03 | 9.17E-01 |
| 2 | rs4491723 | G | A | 3.11E-02 | 4.18E-03 | 1.10E-13 |  | -7.61E-03 | 8.47E-03 | 3.80E-01 |
| 1 | rs4495657 | C | T | 3.28E-02 | 3.76E-03 | 2.40E-18 |  | 5.19E-03 | 7.59E-03 | 5.04E-01 |
| 2 | rs4668354 | G | C | 3.48E-02 | 3.80E-03 | 5.60E-20 |  | 1.40E-03 | 7.79E-03 | 8.61E-01 |
| 6 | rs4716056 | G | A | 2.14E-02 | 3.79E-03 | 1.60E-08 |  | -1.49E-02 | 7.70E-03 | 5.78E-02 |
| 16 | rs4782369 | C | G | 3.00E-02 | 3.93E-03 | 2.10E-14 |  | -1.12E-02 | 8.61E-03 | 2.04E-01 |
| 22 | rs4821798 | C | T | -4.35E-02 | 3.99E-03 | 1.20E-27 |  | -2.27E-02 | 1.00E-02 | 2.65E-02 |
| 13 | rs4886238 | A | G | 3.80E-02 | 3.95E-03 | 6.30E-22 |  | 1.00E-02 | 8.02E-03 | 2.22E-01 |
| 3 | rs507926 | C | T | 2.99E-02 | 4.94E-03 | 1.50E-09 |  | -4.98E-03 | 1.02E-02 | 6.33E-01 |
| 20 | rs536092 | T | C | 2.38E-02 | 3.81E-03 | 3.90E-10 |  | 3.61E-03 | 7.80E-03 | 6.51E-01 |
| 1 | rs55707872 | C | A | 3.54E-02 | 3.98E-03 | 5.70E-19 |  | 1.59E-02 | 8.24E-03 | 5.82E-02 |
| 15 | rs55848327 | A | G | 2.52E-02 | 4.37E-03 | 8.80E-09 |  | 1.92E-02 | 9.65E-03 | 5.14E-02 |
| 5 | rs58279426 | C | T | 6.76E-02 | 3.70E-03 | 1.50E-74 |  | -6.72E-03 | 7.46E-03 | 3.78E-01 |
| 19 | rs60907808 | G | A | -4.44E-02 | 5.26E-03 | 3.20E-17 |  | -1.86E-02 | 1.10E-02 | 9.69E-02 |
| 20 | rs6139074 | C | A | -3.06E-02 | 4.56E-03 | 1.80E-11 |  | -9.37E-03 | 9.90E-03 | 3.55E-01 |
| 10 | rs61870304 | G | A | -3.68E-02 | 6.11E-03 | 1.70E-09 |  | 1.26E-02 | 1.29E-02 | 3.40E-01 |
| 2 | rs62156695 | G | A | -6.41E-02 | 6.04E-03 | 2.70E-26 |  | -8.38E-03 | 1.24E-02 | 5.08E-01 |
| 2 | rs6435156 | T | C | -2.63E-02 | 4.22E-03 | 4.50E-10 |  | -1.19E-02 | 8.66E-03 | 1.78E-01 |
| 8 | rs6470643 | C | A | -2.83E-02 | 4.47E-03 | 2.40E-10 |  | -8.88E-03 | 9.07E-03 | 3.38E-01 |
| 16 | rs6500437 | C | T | -3.55E-02 | 3.97E-03 | 4.60E-19 |  | 7.74E-03 | 8.24E-03 | 3.58E-01 |
| 10 | rs6584351 | G | A | 2.05E-02 | 3.68E-03 | 2.60E-08 |  | 1.78E-02 | 7.49E-03 | 2.01E-02 |
| 1 | rs6667957 | C | T | -2.60E-02 | 3.75E-03 | 4.30E-12 |  | -2.61E-03 | 7.57E-03 | 7.36E-01 |
| 2 | rs6736096 | C | T | 2.01E-02 | 3.68E-03 | 4.70E-08 |  | 1.59E-03 | 7.47E-03 | 8.35E-01 |
| 4 | rs6830848 | T | G | -3.48E-02 | 3.68E-03 | 3.60E-21 |  | -4.95E-03 | 7.66E-03 | 5.27E-01 |
| 6 | rs6912979 | C | T | -2.55E-02 | 4.09E-03 | 4.30E-10 |  | 9.31E-04 | 8.42E-03 | 9.14E-01 |
| 7 | rs6961014 | G | C | -2.90E-02 | 4.51E-03 | 1.20E-10 |  | -1.10E-03 | 9.22E-03 | 9.07E-01 |
| 10 | rs7087644 | G | A | -8.39E-02 | 9.31E-03 | 2.00E-19 |  | -3.82E-03 | 1.85E-02 | 8.40E-01 |
| 11 | rs7125555 | T | C | -2.76E-02 | 3.69E-03 | 6.50E-14 |  | -8.64E-03 | 7.55E-03 | 2.63E-01 |
| 1 | rs72708144 | C | T | 5.88E-02 | 9.21E-03 | 1.70E-10 |  | 1.62E-02 | 2.02E-02 | 4.32E-01 |
| 5 | rs72814771 | G | T | 4.94E-02 | 6.25E-03 | 2.90E-15 |  | 7.89E-03 | 1.23E-02 | 5.30E-01 |
| 2 | rs72827480 | C | T | 2.71E-02 | 3.76E-03 | 6.00E-13 |  | -7.31E-03 | 7.88E-03 | 3.65E-01 |
| 10 | rs728900 | A | T | -3.17E-02 | 3.76E-03 | 3.20E-17 |  | -1.89E-02 | 7.61E-03 | 1.53E-02 |
| 19 | rs73037453 | T | C | -2.64E-02 | 4.69E-03 | 1.70E-08 |  | -2.22E-02 | 9.67E-03 | 2.47E-02 |
| 20 | rs746748 | T | C | 4.74E-02 | 7.29E-03 | 7.50E-11 |  | -4.98E-03 | 1.53E-02 | 7.50E-01 |
| 10 | rs74701710 | A | G | -4.83E-02 | 8.04E-03 | 1.80E-09 |  | 8.80E-04 | 1.62E-02 | 9.58E-01 |
| 7 | rs74742883 | G | T | -3.11E-02 | 4.45E-03 | 3.00E-12 |  | -3.00E-03 | 9.00E-03 | 7.44E-01 |
| 2 | rs7589040 | T | C | -3.15E-02 | 4.50E-03 | 2.40E-12 |  | -1.72E-02 | 8.80E-03 | 5.59E-02 |
| 4 | rs7661090 | T | C | -3.53E-02 | 5.96E-03 | 3.20E-09 |  | 1.66E-02 | 1.19E-02 | 1.72E-01 |
| 2 | rs76928871 | G | A | 4.30E-02 | 4.69E-03 | 4.30E-20 |  | 2.17E-02 | 9.58E-03 | 2.65E-02 |
| 7 | rs7778113 | T | G | 2.80E-02 | 3.92E-03 | 9.40E-13 |  | 1.31E-02 | 8.90E-03 | 1.50E-01 |
| 8 | rs77952879 | C | G | -4.21E-02 | 6.95E-03 | 1.30E-09 |  | 1.88E-02 | 1.56E-02 | 2.39E-01 |
| 6 | rs78080415 | C | T | 2.98E-02 | 5.25E-03 | 1.30E-08 |  | 7.58E-03 | 1.06E-02 | 4.84E-01 |
| 8 | rs7827991 | A | C | 4.17E-02 | 6.88E-03 | 1.30E-09 |  | 1.06E-02 | 1.40E-02 | 4.60E-01 |
| 2 | rs78385274 | C | G | -5.94E-02 | 6.00E-03 | 4.60E-23 |  | 7.35E-03 | 1.15E-02 | 5.32E-01 |
| 8 | rs7845046 | A | T | -4.92E-02 | 8.38E-03 | 4.40E-09 |  | 1.17E-02 | 1.71E-02 | 5.03E-01 |
| 16 | rs8045589 | T | A | -2.26E-02 | 3.70E-03 | 9.70E-10 |  | 7.58E-03 | 7.51E-03 | 3.23E-01 |
| 2 | rs809673 | G | A | -4.17E-02 | 3.77E-03 | 1.70E-28 |  | 1.50E-02 | 7.83E-03 | 6.03E-02 |
| 15 | rs888368 | G | A | 2.22E-02 | 3.68E-03 | 1.70E-09 |  | 3.78E-03 | 7.53E-03 | 6.23E-01 |
| 4 | rs9307242 | C | T | -2.72E-02 | 3.82E-03 | 1.20E-12 |  | 4.64E-03 | 7.81E-03 | 5.62E-01 |
| 5 | rs9313736 | A | G | -3.14E-02 | 3.77E-03 | 8.00E-17 |  | -8.44E-03 | 7.68E-03 | 2.82E-01 |
| 1 | rs9438982 | A | C | -4.71E-02 | 3.95E-03 | 7.40E-33 |  | -2.52E-03 | 7.89E-03 | 7.55E-01 |
| 22 | rs9607474 | T | C | -3.24E-02 | 5.73E-03 | 1.50E-08 |  | -1.68E-02 | 1.40E-02 | 2.41E-01 |
| 22 | rs9613667 | C | A | -2.32E-02 | 3.88E-03 | 2.00E-09 |  | 1.48E-02 | 9.80E-03 | 1.39E-01 |
| 15 | rs9788714 | A | G | -4.29E-02 | 3.81E-03 | 1.80E-29 |  | -1.01E-02 | 7.79E-03 | 2.03E-01 |
| 3 | rs9818740 | A | G | -2.78E-02 | 4.14E-03 | 1.70E-11 |  | 2.15E-03 | 8.51E-03 | 8.05E-01 |
| 17 | rs9915489 | T | A | 4.36E-02 | 3.91E-03 | 7.20E-29 |  | 6.47E-03 | 8.00E-03 | 4.29E-01 |

Supplementary 18

| CHR | SNP | EA | OA | Age at last live birth | | |  | Bone mineral density | | |
| --- | --- | --- | --- | --- | --- | --- | --- | --- | --- | --- |
|  |  |  |  | β | SE | P |  | β | SE | P |
| 9 | rs10964737 | C | T | 8.63E-02 | 1.56E-02 | 2.90E-08 |  | 3.81E-04 | 9.52E-03 | 9.68E-01 |
| 10 | rs12250380 | G | A | 2.02E-02 | 3.44E-03 | 4.20E-09 |  | 5.04E-04 | 2.09E-03 | 8.10E-01 |
| 6 | rs1267490 | A | G | -2.44E-02 | 4.35E-03 | 2.10E-08 |  | -3.32E-04 | 2.67E-03 | 9.01E-01 |
| 20 | rs2208082 | A | G | 1.91E-02 | 3.44E-03 | 2.90E-08 |  | -4.27E-03 | 2.09E-03 | 4.07E-02 |
| 2 | rs359253 | A | G | -2.23E-02 | 3.52E-03 | 2.40E-10 |  | 4.04E-03 | 2.15E-03 | 6.00E-02 |
| 3 | rs6446187 | A | C | -2.92E-02 | 3.38E-03 | 6.20E-18 |  | 1.86E-02 | 2.05E-03 | 1.57E-19 |

Supplementary 19

| CHR | SNP | EA | OA | Age at last live birth | | |  | Heel bone mineral density | | |
| --- | --- | --- | --- | --- | --- | --- | --- | --- | --- | --- |
|  |  |  |  | β | SE | P |  | β | SE | P |
| 9 | rs10964737 | C | T | 8.63E-02 | 1.56E-02 | 2.90E-08 |  | -1.97E-03 | 8.58E-03 | 9.40E-01 |
| 10 | rs12250380 | G | A | 2.02E-02 | 3.44E-03 | 4.20E-09 |  | 1.87E-03 | 1.87E-03 | 2.60E-01 |
| 6 | rs1267490 | A | G | -2.44E-02 | 4.35E-03 | 2.10E-08 |  | 4.87E-04 | 2.36E-03 | 4.90E-01 |
| 20 | rs2208082 | A | G | 1.91E-02 | 3.44E-03 | 2.90E-08 |  | -3.58E-03 | 1.89E-03 | 5.50E-02 |
| 2 | rs359253 | A | G | -2.23E-02 | 3.52E-03 | 2.40E-10 |  | 3.45E-03 | 1.91E-03 | 1.30E-01 |
| 3 | rs6446187 | A | C | -2.92E-02 | 3.38E-03 | 6.20E-18 |  | 1.77E-02 | 1.85E-03 | 1.50E-19 |

Supplementary 20

| CHR | SNP | EA | OA | Age at last live birth | | |  | Ultradistal forearm bone mineral density | | |
| --- | --- | --- | --- | --- | --- | --- | --- | --- | --- | --- |
|  |  |  |  | β | SE | P |  | β | SE | P |
| 9 | rs10964737 | C | T | 0.086338 | 0.015568 | 2.90E-08 |  | -0.055 | 0.050862 | 0.279557 |
| 10 | rs12250380 | G | A | 0.020191 | 0.003437 | 4.20E-09 |  | -0.01458 | 0.010885 | 0.180335 |
| 6 | rs1267490 | A | G | -0.02438 | 0.004353 | 2.10E-08 |  | -0.00842 | 0.01411 | 0.550843 |
| 20 | rs2208082 | A | G | 0.01906 | 0.003437 | 2.90E-08 |  | 0.005489 | 0.010725 | 0.608823 |
| 2 | rs359253 | A | G | -0.02229 | 0.003521 | 2.40E-10 |  | 0.003488 | 0.011157 | 0.754583 |
| 3 | rs6446187 | A | C | -0.02919 | 0.003382 | 6.20E-18 |  | 0.004539 | 0.010623 | 0.669157 |

Supplementary 21

| CHR | SNP | EA | OA | Age at last live birth | | |  | Lumbar spine bone mineral density | | |
| --- | --- | --- | --- | --- | --- | --- | --- | --- | --- | --- |
|  |  |  |  | β | SE | P |  | β | SE | P |
| 9 | rs10964737 | C | T | 0.086338 | 0.015568 | 2.90E-08 |  | -0.00079 | 0.044662 | 0.986184 |
| 10 | rs12250380 | G | A | 0.020191 | 0.003437 | 4.20E-09 |  | 0.001473 | 0.00886 | 0.870996 |
| 6 | rs1267490 | A | G | -0.02438 | 0.004353 | 2.10E-08 |  | 0.01461 | 0.011183 | 0.201968 |
| 20 | rs2208082 | A | G | 0.01906 | 0.003437 | 2.90E-08 |  | -0.00068 | 0.008825 | 0.940428 |
| 2 | rs359253 | A | G | -0.02229 | 0.003521 | 2.40E-10 |  | -0.01338 | 0.009067 | 0.149539 |
| 3 | rs6446187 | A | C | -0.02919 | 0.003382 | 6.20E-18 |  | -5.90E-05 | 0.008939 | 0.994841 |

Supplementary 22

| CHR | SNP | EA | OA | Age at last live birth | | |  | Femoral neck bone mineral density | | |
| --- | --- | --- | --- | --- | --- | --- | --- | --- | --- | --- |
|  |  |  |  | β | SE | P |  | β | SE | P |
| 9 | rs10964737 | C | T | 0.086338 | 0.015568 | 2.90E-08 |  | 0.018422 | 0.038041 | 0.635635 |
| 10 | rs12250380 | G | A | 0.020191 | 0.003437 | 4.20E-09 |  | 0.006157 | 0.007613 | 0.428699 |
| 6 | rs1267490 | A | G | -0.02438 | 0.004353 | 2.10E-08 |  | -0.00013 | 0.00958 | 0.989049 |
| 20 | rs2208082 | A | G | 0.01906 | 0.003437 | 2.90E-08 |  | -0.0015 | 0.007587 | 0.846612 |
| 2 | rs359253 | A | G | -0.02229 | 0.003521 | 2.40E-10 |  | 0.002788 | 0.00778 | 0.725884 |
| 3 | rs6446187 | A | C | -0.02919 | 0.003382 | 6.20E-18 |  | -0.00542 | 0.007642 | 0.487991 |

1. European-descent individuals [↑](#footnote-ref-0)
2. Mixed-descent individuals [↑](#footnote-ref-1)
